# Factors affecting women’s access to primary care in the United States since the Affordable Care Act: A mixed-methods systematic review

**DOI:** 10.1371/journal.pone.0314620

**Published:** 2024-12-19

**Authors:** Allison Gilchrist, Gunasekara Vidana Mestrige Chamath Fernando, Paula Holland, Faraz Ahmed

**Affiliations:** 1 Division of Health Research, Faculty of Health and Medicine, Lancaster University, Lancaster, Lancashire, United Kingdom; 2 School of Nursing, College of Health & Social Services, San Francisco State University, San Francisco, California, United States of America; 3 Department of Family Medicine, Faculty of Medical Sciences, University of Sri Jayewardenepura, Nugegoda, Sri Lanka; University of Pittsburgh Medical Centre, UNITED STATES OF AMERICA

## Abstract

**Background:**

In the United States, the Affordable Care Act (ACA) expanded public and private health coverage, increased health insurance affordability, reduced healthcare costs, and improved healthcare quality for many. Despite ACA provisions, lack of insurance and other factors continue to affect working-age women’s access to primary care services.

**Methods:**

We conducted a mixed-method systematic review to identify factors that affect women’s access to primary care services since the ACA. In January 2021, MEDLINE, CINAHL, PsycINFO, and Web of Science were searched from 2010 to 2021 and an updated search was conducted in October 2023. We included 26 quantitative and qualitative studies reporting determinants, barriers and facilitators of women’s primary care access for women (18 to 64 years). Studies reporting measures of potential access, such as health insurance, and measures of realized access—healthcare service utilization, were included. The Mixed-Methods Appraisal Tool (2018) was used to rate the quality of studies. Andersen’s Behavioral Model of Health Services Use guided the narrative synthesis.

**Findings:**

We found consistent evidence that ACA provisions expanding state Medicaid eligibility led to improved insurance coverage, especially for lower-income groups. We found mixed evidence of associations between individual-level determinants, such as age, education, race/ethnicity, income, and different measures of access. Limited qualitative evidence suggests that insurance coverage, low-cost care, positive patient-provider relationships, social support, and translation services enhance access for immigrants and refugees. Barriers include lack of coverage, high healthcare costs, culturally unresponsive healthcare services, poor patient-provider relationships, and transportation issues.

**Conclusion:**

Adoption of ACA’s expanded Medicaid eligibility criteria would expand insurance coverage to women living in non-expansion states. Innovative healthcare policies, programs, and interventions at the federal, state, and local levels are needed. Suggested strategies include interventions expanding primary healthcare service availability and patient navigation services, and promotion of health literacy, culturally sensitive services, and provider bias education/training.

## Introduction

Women living in the United States (U.S.) face significant challenges accessing healthcare and experience poorer health outcomes compared to women living in other high-income countries [1, 2]. U.S. women have the worst access to healthcare and insurance, the highest rates of delayed or missed healthcare due to cost or affordability issues, the most avoidable deaths, and the poorest life expectancy compared to women living in 13 other high-income countries [2]. The Affordable Care Act (ACA) provisions significantly improved women’s access to healthcare services by expanding coverage, making healthcare more affordable, and ensuring that women are no longer denied coverage due to pre-existing conditions such as pregnancy and are covered for essential services such as preventive or reproductive healthcare. Nevertheless, barriers to access, coverage and affordability persist for women [3, 4]. Despite gains made by the ACA, 10% (9.5 million) of all women (19–64 years) were uninsured in 2022 [5]. U.S. women continue to be disproportionately impacted by issues related to health coverage, affordability and financial costs [6, 7]. A systematic review of factors that affect U.S. working-age women’s access to primary care services since the implementation of the ACA is vital to highlight current research gaps, and to inform directions for the future healthcare policies and practices needed to expand healthcare access for women.

Adult women face many unique sex and gender-specific barriers to accessing primary care services in the U.S. [8]. Women in the U.S. are less likely to be uninsured than men, and are more likely to face barriers to obtaining healthcare, due to affordability or delays in receiving care [7]. In 2021, men were more likely to be uninsured compared to non-elderly women (14% versus 11%), however, more women were covered by the federal-state Medicaid program for low-income individuals compared to men (18% versus 14%) [9]. Women are more likely to qualify for Medicaid as they earn less than men and are more likely to meet Medicaid’s eligibility criteria which include having a disability, being a parent of children under 18, being pregnant, or being more than 65 years [9]. Women were also more likely to have multiple chronic diseases compared to men (28.4% and 25.9% respectively) in 2018 [10]. In 2020, more working-age women reported visiting a provider in the past two years and having a usual source of care compared to men [11]. However, more women (27%) (18 to 64 years) in 2022 reported difficulties paying medical bills compared to men (23%) in the past year [6]. In 2020, U.S. women earned 84% of men as measured by the median hourly earnings of both full-time and part-time workers [12]. This persistent gender wage gap indicates women are less likely than men to be able to pay healthcare costs. Many other non-financial barriers also affect working-age women’s access to primary care, including age, disability status, geographic location, income, racial or ethnic group, geographic location, sexual orientation, and socioeconomic status [8].

Previous systematic reviews suggest that insurance coverage is associated with improved access to health services and better health outcomes in adult U.S. populations [13–15]. Few U.S.-based systematic reviews have examined factors that impact adult women’s access to healthcare before or during the ACA era. Most focus on sub-groups such as immigrant women [16–19], the effect of ACA provisions on women’s access to reproductive care [17, 20–22], and breast or cervical cancer screening [23–29]. A recent literature review suggests that while the ACA may have led to an overall improvement in health insurance coverage, healthcare access, affordability, contraceptive use, mental health care, perinatal outcomes, and use of preventive services for women, multiple barriers to access still exist, placing women at higher risk of poor health outcomes [30]. The authors cite methodological limitations as a systematic review was not conducted, and the review only included a few studies [30].

Despite the gains of the ACA, working-age women continue to experience barriers to healthcare access. A search of the literature revealed the lack of a synthesis of current empirical evidence about factors that impact working-age women’s access to primary care following the ACA. To address this gap, we conducted a mixed-method systematic review to provide a synthesis of evidence about what is known about individual and contextual factors that impact women’s access to primary care after implementation of the ACA, applying Andersen’s Behavioral Model of Health Service Utilization [31] as a framework. Study objectives included: i) identifying individual and contextual factors that impact access to primary care services among adult working-age women in the ACA era, and assessing the consistency of and quality of evidence supporting associations between each factor and measures of access; ii) exploring barriers and facilitators that adult working-age women experience accessing and using primary care services.

## Materials and methods

We adopted a Mixed-method systematic review (MMSR) design as findings from various quantitative, qualitative, or mixed-methods studies can provide a more in-depth understanding of evidence [32, 33]. A narrative synthesis approach was chosen as an appropriate method for synthesizing diverse evidence applying a theoretical framework [34]. Mixed evidence enhances the relevance of findings for different stakeholders.

### Protocol and registration

This mixed-method systematic review was performed and reported according to Preferred Reporting Items for Systematic Reviews and Meta-Analyses (PRISMA) Guidelines [35] (S1 File). The protocol was registered with Prospero with registration number CRD42021265314 [36]. Ethical approval was not required as this was a mixed-method systematic review of published literature.

### Search strategy and eligibility criteria

#### Data sources and searches

We searched MEDLINE, CINAHL, and PsycINFO via EBSCOHost and Web of Science for peer-reviewed studies published in English from 2010 to January 2021. Search strategies were developed in consultation with a library information specialist. For search terms, the main subject domains, Medical Subject Heading (MeSH) or free text terms for “women,” “primary care,” “access and utilization,” and “United States” were combined with the Boolean operator ‘AND’ in final searches. The search strategy was initially developed in MEDLINE and corresponding searches were conducted in CINAHL, PsycINFO and Web of Science databases (S2 File). We conducted database searches on 25 and 26 January 2021. An updated search in MEDLINE, CINAHL, PsycINFO and Web of Science using the same search strategies was conducted on 10 October 2023.

#### Study selection

We applied several inclusion criteria in selecting studies. First, empirical studies had to include findings relating to adult working-age (18 to 64 years) women. Second, U.S.-based studies that examined factors associated with providing formal, face-to-face primary health services to women were included. Third, studies had to report outcome measures of potential or realized access, including 1) health insurance; 2) usual source of care/regular primary care provider; 3) healthcare service utilization; 4) routine preventive health screenings and well visits; and 5) unmet healthcare needs. Fourth, empirical studies had to employ a quantitative, qualitative, or mixed methods design and be published in English between 2010 and 2023. We excluded studies conducted in institutional settings, studies that reported outcome measures for specialist or tertiary health services or disease-specific care, and studies reporting utilization of reproductive, sexual health services, or breast, cervical, or colorectal cancer screening services for women. Book chapters, conference abstracts, editorial commentary or opinion papers, or gray literature reporting non-peer-reviewed empirical research were excluded. A detailed summary of inclusion/exclusion criteria is outlined in S1 Table.

Database search results were uploaded to Endnote -software and then screened for duplicates, which were removed according to PRISMA guidelines [35]. The first reviewer (AG) independently screened all the titles, reviewed abstracts of potentially eligible studies and reviewed all studies eligible for a full-text review. The second reviewer (CF) independently screened a 10% random sample of abstracts and titles, and a 10% random sample of full-text studies to ensure study criteria were applied consistently. Disagreements were resolved between the first and second reviewers. The third reviewer (FA) arbitrated disagreements if the first and second reviewers (AG, CF) could not reach a consensus. Searches were supplemented with backwards and forward searches of all references of included studies.

### Data extraction and quality assessment

An extraction tool was developed by adapting the Joanna Briggs Institute (JBI) Mixed Methods data extraction tool [37], and the extraction tool was piloted with ten studies. We extracted primary data about the authors, title, journal, study design, study aims/objectives, conceptual model or framework, population characteristics, sample size, sample characteristics such as year data collected and data sources, geographic setting, inclusion/exclusion criteria, data collection methods, measurement tools, relevant statistical findings of associations between determinants (independent variables) and outcome measures (dependent variables) of interest for quantitative studies or themes developed in qualitative studies. The first reviewer (AG) extracted data for all eligible studies using the revised data extraction form and summarized the data, and the second reviewer (CF) independently reviewed 25% of the data extraction forms for accuracy. The completed data extraction form is available in S3 File.

The quality of quantitative and qualitative studies was assessed using the Mixed Methods Appraisal Tool (MMAT) (2018) [38]. The MMAT (2018) was considered a suitable tool, as the content validity of the MMAT has been substantiated; each domain was developed from consultations, relevant literature and workshops and evidence suggests the tool has ecological validity with transferability of findings to real-world settings [39, 40]. The tool applied the following scoring: 100% (5), 80% (4), 60% (3), 40% (2), or 20% (1) quality criteria met. We evaluated quantitative non-randomized designs based on several criteria, including sample representativeness, appropriate exposure and outcomes measurements, complete outcome data, whether confounders were accounted for, and whether the intervention or exposure was administered as intended. Qualitative studies were evaluated based on the qualitative approach, data collection methods, findings, interpretation, and coherence of findings. Two reviewers (AG and CF) independently assessed the methodological quality of all eligible empirical studies using the MMAT (2018) and assigned an overall score for quality criteria met for each study [38]. Any disagreements in ratings were resolved after discussion. No studies were excluded based on study quality, as the mixed-method systematic review was exploratory.

### Conceptual model

This review used Andersen’s Behavioral Model of Health Services Use [31] as a framework to guide the synthesis of findings. Andersen’s model has been applied extensively in empirical studies to examine determinants, barriers, and facilitators that influence healthcare access in different populations [41]. The model incorporates individual and contextual determinants of healthcare access. These include predisposing factors such as sociodemographic characteristics, enabling factors such as income, insurance or public policies, and healthcare financing, and need characteristics such as individual perceived need and evaluated need for healthcare or population health indices. These determinants predict personal health practices, patterns of healthcare utilization, perceived and evaluated health status, and perceived satisfaction with care. Outcomes in Andersen’s model include perceived health status, evaluated health status, and consumer satisfaction. Access is defined as either potential access (for example, insurance coverage, usual source of care) or utilization of health care services (for example, ambulatory care services for acute, chronic problems, or preventive health care) [31].

### Data synthesis and analysis

We adopted a narrative synthesis approach because of the heterogeneity of research methodologies, focal populations, and variability of outcomes reported in the studies [34]. Andersen’s model [31] was used to organize and report the findings. In the first step, we applied an iterative process and, narratively summarized, synthesized, and presented findings from 23 quantitative studies. Patterns, similarities, differences, and relationships between studies were explored, and the robustness of the synthesis was assessed [34]. Statistically significant results were identified, categorized as positive, negative, or no relationship, and grouped under the Andersen model domains in a tabular format. In the second step, we conducted a thematic analysis of the findings and discussion sections of the included qualitative studies. Major themes relating to facilitators and barriers to access were identified and grouped according to Andersen’s domains. Data from three eligible qualitative studies were extracted, coded then organized into themes using NVivo 12, a Computer-assisted qualitative data analysis software program. In the final step, to address the study objectives, findings from quantitative and qualitative studies were integrated and synthesized to map the current evidence base regarding determinants, facilitators, and barriers to women’s access to health care. Relationships within and between studies and differences across studies were explored.

## Results

**Fig 1:**
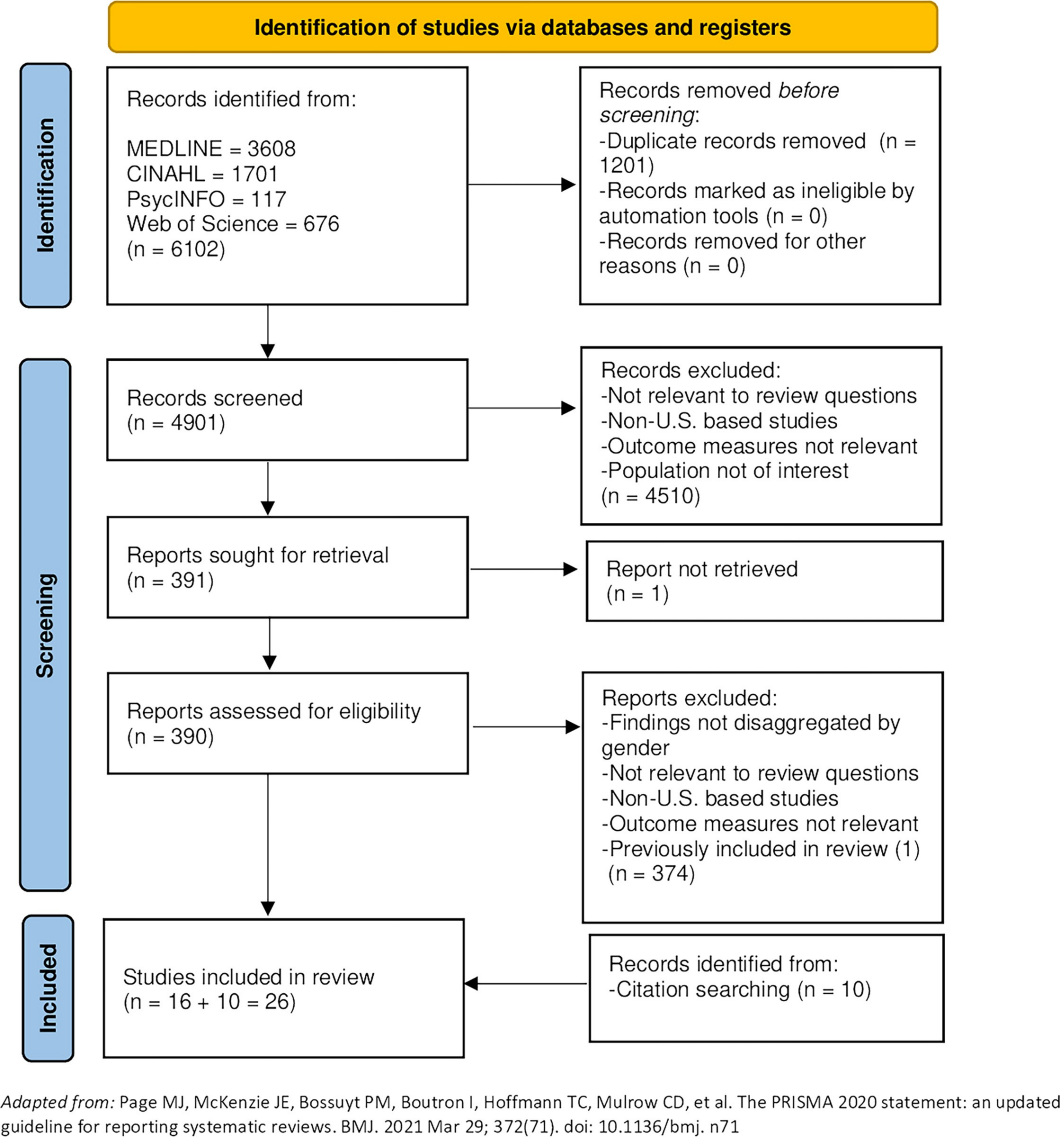
PRISMA flow diagram of the systematic review search process.

Our search strategy resulted in 4901 abstracts after the removal of duplicates. We identified 391 studies for full review, and 16 were eligible for inclusion. Backwards and forward searching of included studies and relevant systematic reviews identified ten additional papers. Twenty-six studies were included in the review [42–67]. Search results are summarized in the PRISMA flowchart diagram of the systematic review search process (Fig 1). This mixed-method review is reported according to the PRISMA 2020 Checklist (S1 File) [35].

### Study characteristics

Most studies were quantitative (23/26, 88%) [42–49, 51–56, 58–60, 62–67], and used a cross-sectional design (11/23, 48%), a panel or interrupted time series without a control group design (2/23, 9%), or a difference-in-difference (panel or repeated cross-sectional survey data) design (10/23, 43%). The majority were secondary analyses of national household survey datasets (16/23, 70%). Three studies (3/26, 12%) applied a qualitative design [50, 57, 61]. Most studies were nationally based, or conducted in selected Medicaid expansion and non-expansion states (18/26, 69%), occurred in mixed urban and rural settings (20/26, 77%), and targeted women (19/26, 73%) between 18 to 64 years. Studies were published between 2014–2021, with the majority (24/26, 92%) published between 2016–2021. The quality score of studies assessed using the MMAT [38] was 5/5 (13/26, 50%), 4/5 (9/26, 35%), and 3/5 (4/26, 15%) respectively (S4 File). Studies were not excluded based on an assessment of poor quality. Table 1 reports the study design, sample characteristics, data sources, and quality assessment scores of all included studies.

**Table 1:** Study design, sample characteristics, quality assessment, and key findings.

First Author, Year	Study design/ methodology	Purpose	Sample characteristics and size, setting	Data source/Year	Quality Score*	Key Findings
Ahad, 2019	Cross-sectional survey	Examined the association between immigrant status, health status, health literacy and barriers to access, and having a regular healthcare provider in African immigrants compared to African-American women	African American and African immigrant women (18–64 years) living in an urban setting in Utah, (N = 165); 87 African American women, 78 African immigrants	Coalition for a Healthier Community for Utah Women and Girls’ study (2012–2018)	4/5, 80%	Compared to U.S.-born African American women, African immigrant women had significantly lower odds of having a regular health provider and were more likely to be uninsured (19.23% versus 11.49%)
Dai, 2019	Cross-sectional telephone survey	Assessed access to healthcare, health status, and preventive health behaviors by sexual orientation stratified by gender and age group (50–64 years or 65 years or older) in selected geographic regions in the U.S.	Adults (50–64 years) in select U.S. regions, All adults (N = 350,778)	Behavioral Risk Factor Surveillance System (BRFSS) (2014–2016)	5/5, 100%	Older women (50–64 years) who reported their sexual orientation as other had significantly increased odds of not having insurance or a regular health provider compared to heterosexual peers. Lesbians had increased odds of having received a flu shot in the past year compared to straight peers.
DiPietro Mager, 2021	Cross-sectional survey	Examined healthcare access measures, individual and system-level barriers, and characteristics associated with routine healthcare service use among reproductive-age women living in a rural maternity care desert	Women (18–45 years) living in Hardin County, a rural county in northwest Ohio, (N = 315)	Cross-sectional survey (February-May 2019)	3/5, 60%	In a sample of women of reproductive age living in a rural area, 11.3% had no health insurance, 14.4% had no usual source of care, 22.8% had not had a routine check-up in the past 12 months, and 53% reported at least one barrier to healthcare access. Having had a routine check-up in the past 12 months was associated with lower odds of barriers.
Early, (2018)	Cross-sectional survey	Assessed how ACA’s Medicaid expansion in California impacted health insurance coverage and receipt of healthcare services including contraceptive counseling and contraceptive prescriptions in low-income women	Low-income (<138% FPL) reproductive-age women, (18–44 years) (N = 4,567)	The California Health Interview Survey (CHIS) (2013–2016) (2013 versus 2014–2016)	4/5, 80%	From 2013 to 2016, the uninsured rate of low-income women decreased significantly from 29% to 11%, and enrollment in Medicaid increased significantly from 37% to 67%. Pre to post-expansion, insignificant changes in a usual source of care (83% versus 77%), and an ability to obtain needed healthcare (85% versus 82%) were reported.
Farietta, 2018	Cross-sectional telephone survey	Evaluated changes in healthcare utilization and unmet needs for healthcare among low-income reproductive-age women following the ACA’s Medicaid expansion in 2014	Low-income (≤138% PL) women (19–44 years) newly eligible for Medicaid after expansion in January 2014), 2012 survey (N = 489); 2015 survey (N = 1273)	Ohio Medicaid Assessment Survey (OMAS) (2012, 2015)	5/5, 100%	In 2015 compared to 2012, low-income reproductive-age women reported significantly lower odds of having unmet needs for mental health, dental care, vision care, or prescriptions. No significant differences in the odds of visiting a doctor in the last year or a usual source of care in reproductive-age women were reported.
Johnson, 2020	Cross-sectional survey	Examined unmet healthcare needs among middle-aged women (50–64 years) in the U.S. according to level of psychological distress	Women (50–64 years), (N = 8,838)	National Health Interview Survey (NHIS), (2015–2016)	4/5, 80%	Women reporting moderate or severe psychological stress had two to five times higher odds of delayed care, and two to twenty times higher odds of foregone care compared to women reporting no psychological distress.
Jones, 2016	Cross-sectional survey	Assessed changes in rates of insurance and differences in insurance rates according to the state’s Medicaid expansion status in the context of ACA healthcare reform	Women (18–39 years), (N = 8000); 2012 (N = 4593); 2015 (N = 3407)	Two national surveys developed by the Guttmacher Institute and administered by GfK (2012 versus 2015)	4/5, 80%	Post-expansion increased Medicaid and private insurance coverage, rates of uninsured women decreased from 18.9% in 2012 to 11.5% in 2015. However, several groups, including U.S. and foreign-born Latinas, experienced no significant decrease in uninsured rates in Medicaid expansion states. Low-income women’s uninsured rates decreased from 38% to 15%, mainly due to expanded Medicaid coverage.
Massetti, 2017	Cross-sectional survey	Examined whether mental health problems are associated with increased risk factors for cancer and lower rates of health-protective behaviors including cancer screening services in young adults	Young adults (18–39 years), (N = 90,821); Men (N = 41,906); Women (N = 48,915)	BRFSS (2014)	5/5, 100%	Women with mental health problems compared to those with none were significantly less likely to have received a health check-up in the last two years (82.2% versus 79.5%, p < 0.001). Rates of healthcare coverage and receipt of influenza vaccine in the past 12 months were not significantly different for women with mental health issues compared to those without mental health issues.
Pazol, 2017	Cross-sectional survey	Assessed utilization of recommended key preventive health services in women of reproductive age according to age, race/ethnicity, income levels, and continuity of insurance coverage in 2013	Women (18–44 years), (N = 8,244)	NHIS (2013)	3/5, 60%	Women in the highest family income category (>400% FPL compared to ≤ 138% FPL) and women with continuous insurance coverage over the last year (compared with those with gaps in insurance or no insurance) had the highest rates of blood pressure checks by a provider and receipt of influenza vaccine in the past year. Women in the lowest family income category (≤ 138% FPL) had the lowest receipt of BP checks and influenza vaccine.
Seo, 2019	Cross-sectional survey	Evaluated factors that impact healthcare utilization among Foreign-Born Asian Immigrant (FBAI) women compared to Native-Born (non-Hispanic) White American (NBWA) women	Women (18–64 years), FBAI women (N = 1,021); NBWA (N = 7,086)	CHIS (2014–2015)	5/5, 100%	Compared to NBWA, FBAI women reported they were significantly less likely to have visited a doctor in the past year and were less likely to report a usual source of care. For FBAI, significant predictors of at least one visit to the doctor in the past year included having health insurance, a source of usual care, being diagnosed with chronic disease, and a high school education.
SteelFisher, 2019	Cross-sectional survey	Examined experiences of women of different ethnic groups and lesbian, gay, bisexual, transgender, and queer (LGBTQ) individuals regarding discrimination and harassment in healthcare settings	Women (18–64 years), (N = 1596)	SSRS administered a national telephone survey (January-April 2017)	3/5, 60%	Eighteen percent of women reported discrimination in healthcare settings. Black, Latina and Native American women had increased odds of reporting gender discrimination in healthcare compared to white women. Latina women had higher odds of avoiding healthcare due to discrimination concerns compared to white women.
Daw, 2019	Interrupted time series without a control group	Assessed the association between the ACA’s major coverage expansions and insurance coverage, access to a usual source of care, and barriers related to the cost of care in women of reproductive age, including pregnant women	Reproductive-age women (18–44 years), (N = 128,352); 2010–2013 (N = 74,175); 2014–2015 (N = 54,177); Pregnant women (N = 2,179); 2010–2013 (N = 1,263); 2014–2015 (N = 916)	NHIS, (2010–2013, 2014–2016)	5/5, 100%	Among women of reproductive age, the ACA was associated with a 3.6 percentage-point increase in Medicaid enrollment, a 7.4 percentage-point decrease in the probability of uninsurance, a 2.4 percentage-point decrease in not having a usual source of care, and a 1.5 percentage-point decrease in cost being a barrier to healthcare. Overall the ACA was associated with expanded insurance coverage and access to healthcare for reproductive-aged women, particularly lower-income women.
Lee, 2020	Panel Survey (overlapping panel design)	Examined differences by provider type in past-year provider visit rates for reproductive-aged women to determine rural-urban differences in access to healthcare	Reproductive-age women (18–44 years), (N = 37,026)	Medical Expenditure Panel Survey (MEPS) (2010–2015) linked to the Area Health Resource File	5/5, 100%	Overall the study found no rural-urban differences in past-year visits with healthcare providers, but there were differences by provider type. Women living in rural areas had higher past-year visit rates to family medicine physicians (24.3% versus 20.9%) and nurse practitioners/physician assistants (NPs/PAs) (24.6% versus 16.1%) and lower past-year visit rates to obstetrician-gynecologists (OB-GYN) (23.3% versus 26.6%)
Chen, 2020	Difference-in-differences design	Assessed changes in insurance coverage, healthcare spending, and utilization in low-income women in Medicaid expansion compared to non-expansion states	Low-income (<138% FPL) reproductive-age women (19–44 years) (N = 1,124) **Sub-sample** Low-income reproductive-age women who were uninsured in 2013 (N = 149)	MEPS (2013–2014) (2013 versus 2014)	4/5, 80%	Medicaid expansion was associated with significantly more uninsured women gaining Medicaid in expansion states (38.7%) compared to non-expansion states (19.5%) (P = .01). Black (13.4%) and Hispanic women (12.0%) were significantly less likely to gain Medicaid coverage compared to white women (42.8%)
Courtemanche, 2019	Difference-in-differences design	Estimated the impact of ACA’s major reforms (Medicaid expansion, insurance market reforms, and subsidized Marketplace plans) on inequalities in insurance coverage three years after implementation	Adults (19–64 years), All adults (N = 10,537,667); Women (N = 5,473,836)	American Community Survey (ACS) (2011–2016) (2011–2013 versus 2014–2016)	4/5, 80%	The fully implemented ACA led to a 43% reduction in the coverage gap across income groups after three years. The ACA also led to a decrease in health insurance inequalities across age groups (36%), racial and ethnic groups (23%), and marital status (46%)
Johnston, 2018	Difference-in-differences design (two-way fixed effects)	Examined the impact of Medicaid expansions (including pre-ACA eligibility levels, parental status, and presence of a state Medicaid family planning waiver) on insurance coverage and access to healthcare in low-income reproductive-age women	Low-income (<138% FPL), non-pregnant reproductive-age women (19–44 years), (Sample size ranged from N = 24,955–25,816 according to the dependent variable)	BRSS (2012–2015) (2012–2013 versus 2014–2015)	5/5, 100%	ACA Medicaid expansions reduced uninsurance among reproductive-age low-income women by 13.2 percentage points and reduced barriers to care due to cost by 3.8 percentage points. Uninsurance decreased by 27.4 percentage points, and the likelihood of not having a personal doctor was reduced by 13.3 percentage points in women without dependent children.
Lee, 2018	Difference-in-differences design	Evaluated the impact of Medicaid expansion on health insurance coverage and affordability of healthcare in women according to income level	Women (19–64 years), (N = estimated 95,610,990 women nationally)	NHIS (2010–2013, 2015) (2010–2013 versus 2015)	4/5, 80%	Post-ACA Expansion (2015), rates of uninsurance decreased across all income groups compared to pre-expansion (2010–2013). The lowest income group (≤ 138% FPL) had significantly decreased odds of being uninsured compared to the highest income group (≥ 400% FPL).
Lee, 2019	Difference-in-differences design	Compared changes in women’s access to healthcare, insurance affordability, and use of preventive services by income level pre- and post-Medicaid expansion	Women (19–64 years), (N = 105,021, representing 41,106,929 women nationally)	NHIS (2011–2017) (2010–2013 versus 2014–2017)	5/5, 100%	Following ACA insurance expansions, low-income women (≤ 138% FPL) found it less challenging to find affordable health insurance and were more likely to have seen or talked to a doctor in the past year, and had blood pressure screening, or cholesterol screening in the past year, compared with higher-income women (≥ 400% FPL).
Margerison, 2020	Difference-in-differences design	Assessed the impact of expanded Medicaid eligibility on preventive healthcare measures and behaviors among low-income women of reproductive age before and after the implementation of ACA’s Medicaid expansion	Low-income (<138% FPL) reproductive-age women (18−44 years), (N = 58,365) in 38 states including DC	BRFSS (2011−2016) (2011−2013 versus 2015−2016)	5/5, 100%	In low-income women, Medicaid expansion was associated with increased insurance coverage, utilization of healthcare, and reductions in healthcare avoidance due to cost. Among married women, expanded Medicaid eligibility led to more significant increases in insurance coverage and healthcare utilization compared to non-married women. Women with dependent children had lower gains in insurance coverage compared to women with no dependent children.
Simon, 2017	Difference-in-differences design	Examined the impact of the ACA expansions on preventive care service utilization, including cancer screenings and immunizations, risky health behaviors, and self-assessed health status in low-income childless adults	Low-income (<100% FPL) childless adults (19–64 years), Medicaid expansion states (N = 74,423); Medicaid non-expansion states (N = 77,140) (Sample size for women varies according to the dependent variable)	BRSS (2010–2015 (2010–2013 versus 2014–2015)	5/5, 100%	ACA Medicaid expansions led to increased insurance coverage and access to care for low-income childless adults and increased rates of utilization of certain preventive healthcare services. Medicaid expansion resulted in a 3.4 percentage point or 5% increase in insurance rates in low-income women.
Sommers, 2014	Difference-in-differences design	Evaluated the rate of enrollment of newly eligible adults after expanded Medicaid eligibility, and the extent to which new enrollment of adults without children was due to extending insurance coverage to uninsured individuals versus replacing private coverage	Low-income (<138% FPL) childless adults (19–64 years); D.C. versus Virginia: Childless adults (N = 35,013); Women (N = 16,098) Connecticut versus the other Northeast States: Adults (N = 109,292); Women (N = 49,878)	1) Monthly Medicaid enrollment statistics in 4 states; 2) ACS (2008–11) (2008–2009 versus 2011)	3/5, 60%	Medicaid expansion in D.C. compared to Virginia increased Medicaid coverage in women (5.3%, p < 0.10). In Connecticut, Medicaid expansion increased coverage significantly (1.5%, p < 0.01) compared to Northeast states. Women in D.C. compared to Virginia had a 3.3% decrease in uninsurance, while women in Connecticut had a 2.8% decrease (p < 0.01) in uninsurance compared to other Northeast states.
Sommers, 2015	Difference-in-differences design/time-series analysis	Estimated national changes in access to healthcare and insurance coverage measures in low-income adults during the ACA’s first two open enrollment periods, and examined differences between Medicaid expansion versus non-expansion states	Low-income adults (18–64 years), (N = 507,055); Low-income women (18–64 years), (N = 240,855)	National Gallup-Healthways Well-Being Index telephone survey (2012–2015) (2012–2013 versus 2014–2015)	4/5, 80%	In 2015, women experienced a decrease in uninsured rates, decreased rates of not having a personal physician, and reduced inability to afford care compared to the pre-ACA trend. Medicaid expansion was associated with significant reductions in uninsured rates, no personal physician, and difficulty accessing medicine among low-income adults.
Wehby, 2018	Difference-in-differences design	Examined the effect of ACA Medicaid expansion on Medicaid enrollment and private coverage and inequalities in Medicaid coverage according to age, gender, and race/ethnicity up to 2015	Low-educated (high school or less) adults (19–64 years) (<138% FPL), (N = 3,137,989); Women (N = 1,438,733)	ACS (2011–2015) (2011–2013 versus 2014–2015)	5/5, 100%	Rates of Medicaid enrollment were higher in eligible women (6.4 percentage points) compared to men (5.8 percentage points). All subgroups stratified by gender and race/ethnicity experienced significant increases in Medicaid enrollment, with Hispanic women experiencing the largest increase in Medicaid coverage (7.5 percentage points). Hispanic women and non-Hispanic black men had the largest decline in uninsurance at 5.1 percentage points.
Greder, 2019	Qualitative—thematic analysis	Explored experiences relating to being healthy, health promotion strategies used, and challenges to and support for health in Latina immigrant women	First-generation Mexican immigrant women (21–47 years) living in 2 rural counties in a Midwestern state (N = 15)	Qualitative interviews (2012)	5/5, 100%	This study reported four thematic areas of interest with ten associated themes. These included 1) meaning associated with being healthy (absence of illness and good with family); 2) health-promoting strategies (activity with family and intentional consumption); 3) health challenges (juggling roles, lack of easily accessible and culturally responsive health care, and unjust housing); and 4) health support (faith is like medicine, healthcare safety net, and women allies).
Luque, 2018	Qualitative—thematic analysis	Explored how different individual and structural factors impact uninsured immigrant Latina women’s access to medical care, preventive screenings, and alternative strategies employed by women seeking medical treatment	Uninsured Latina immigrant women (21–64 years) living in Charleston metropolitan area, South Carolina (N = 30)	Semi-structured interviews (Fall-winter, 2016)	5/5, 100%	Four themes were identified: 1) disease management strategies, 2) cultural factors, 3) health behaviors and coping mechanisms, and 4) facilitators and barriers to healthcare. Facilitators included access to language translation services, low-cost medicines, and social support from various sources. Family and work commitments, the high cost of healthcare services, lack of insurance, and language barriers were barriers to care.
Ross Perfetti, 2019	Qualitative—thematic analysis	Explored Iraqi refugee women’s attitudes about health and preventative care services such as cancer screenings, and cultural and structural mediators of health in the context of women’s lives	Iraqi refugee women (18–64 years) living in Philadelphia (N = 14)	Three focus groups (May-June 2016)	4/5, 80%	Eight themes were identified relating to attitudes about and mediators of health. Three themes relating to barriers to healthcare access included financial barriers, multi‐level problems within hospitals and clinics, and competing priorities. Other themes included, cancer being caused by dangerous environments, embracing biomedical and alternative healing practices, God contributes to healing, physical and mental health being interrelated, and ’prevention is better than cure.’

*Quality score based on a 5-point scale used in the MMAT (2018) developed by Hong et al. (2018).

### Overall synthesis of quantitative evidence

#### Outcome measures

Table 2 summarizes the main outcomes reported in the 23 included quantitative studies. The outcomes reported in quantitative studies relate to measures of potential access (such as health insurance, usual source of care, and other barriers such as cost of health care) and measures of realized access (such as utilization of primary care services and preventive health screenings). Most quantitative studies (20/23, 87%) reported measures of potential access. Measures included insurance coverage and type (14/23, 61%), usual source of care/regular healthcare provider (9/23, 39%), cost or affordability (9/23, 39%), and delayed or foregone care (5/23, 22%). In comparison, fewer studies (11/23, 48%) examined measures of realized access or utilization of healthcare services, such as visits to a doctor or other healthcare professional for a routine checkup or health concerns (10/23, 43%), or receipt of preventive screenings in the past year (6/23, 26%). The operationalized definitions of outcome measures used to measure potential and realized access varied considerably, making it difficult to compare findings across studies.

**Table 2:** Differences and variations in reported indicators of healthcare access in quantitative studies.

Measures of potential access	Empirical studies
**Health insurance**	
Health insurance/has healthcare coverage	[44, 58, 59, 63]
Uninsured/no healthcare coverage	[43, 45–47, 52, 53, 64, 65, 67]
**Type of health insurance**	[43, 44, 46, 48, 53, 64, 67]
**Usual source of care/regular healthcare provider**	
Has a personal doctor/regular healthcare provider	[42, 47, 63]
No personal doctor/healthcare provider/physician	[45, 52, 65]
Usual source of health care	[46, 48, 49]
**Other barriers to access**	
Ability to obtain needed medical care without delay, having timely access to prescriptions	[48]
Avoided doctor or health care because of concerns about gender discrimination/poor treatment	[66]
Barriers to receipt of health services	[47]
Could not /difficulties/problems afford/ing a doctor/medical care/medical bills due to cost/cost barrier to care	[45, 55, 63, 65]
Delayed care or foregone/no receipt of medical care due to cost	[46, 51, 52, 55, 58]
Insurance affordability	[56]
Unmet Need for mental health counseling, prescription drugs	[49]
**Measures of realized access**	
Doctors visit, primary care provider visit, primary care visit	[49, 52, 54, 56, 62]
Routine checkup	[45, 47, 58, 59, 63]
Blood pressure check	[56, 60]
Cholesterol check	[56, 58]
Influenza vaccination	[45, 56, 59, 60, 63]

#### Determinants associated with primary care access and health service utilization

Most quantitative studies examined associations between individual predisposing, enabling, or contextual enabling factors, such as the ACA Medicaid expansion’s impact on access measures. Few studies assessed contextual predisposing factors, individual or contextual need factors, or behaviors. Identified determinants were classified according to Andersen’s framework, however, the following determinants—English proficiency, having children or dependents, immigration status/nativity, the number of births, or family size are not included in the applied Andersen’s model [31]. Several other studies have categorized these determinants as individual predisposing factors according to Andersen’s model [41]. The findings for associations between individual and contextual factors described in the Andersen model and different measures of access and healthcare utilization are reported in S2 Table.

#### Individual predisposing factors

While certain trends were noted with individual predisposing factors such as age, race/ethnicity, and educational level, findings were not consistent across all studies. Several studies reported ACA’s Medicaid expansion was associated with significantly increased insurance coverage rates in uninsured women [53, 55, 67] and Medicaid-insured women [67], across all age groups. Relative to younger women (18–24 years), older women (25–34 years) were more likely to be insured in Medicaid expansion states [53]. One cross-sectional survey conducted in Utah found older age was associated with having a regular provider [42], while another California-based cross-sectional survey found a significant increase in a younger age group (18 to 34 years) having access to a usual source of care by study year (change between 2013 and 2016) [48]. Age was not associated with a routine check-up in the past year post-Medicaid expansion [47], a Nurse Practitioner or Physician’s assistant visit in the past year [54], or with one or more doctor’s visits in the past year [62] or avoidance of a doctor because of concerns about discrimination [66] in working-age women.

Several studies reported associations between race and ethnicity and various measures of access [53–55, 62, 66, 67]. One study of low-educated adults (19–64 years) found significant decreases in uninsurance rates and increased Medicaid coverage across all racial and ethnic groups, with Hispanic women experiencing the highest gains in health coverage and Medicaid in expansion states [67]. Similarly, Lee et al. (2018) found that all groups of adult women experienced decreases in uninsurance, with low-income (≤ 138% FPL) Hispanic women reporting the largest decreases. Another study of reproductive-age women found that while uninsured rates decreased across all groups, both U.S.-born Hispanic and foreign-born Hispanic women had significantly increased odds of being uninsured compared to white women post-Medicaid expansion [53]. In adult women (18–64 years) Hispanic and Native American ethnicity were associated with avoidance of doctors because of concerns about discrimination [66]. Compared to adult non-Hispanic white women, women from other racial/ethnic groups had significantly fewer family physician or nurse practitioner/physician assistant visits in the last year [54, 62].

Evidence regarding the association between educational level, employment, marital status, family dependents, and access measures was mixed. Several studies reported some associations between educational level and various measures of access [42, 48, 49, 53, 54, 62, 66], however, other studies found no such association [43, 47]. Full-time employment status post-Medicaid expansion was associated with lower levels of uninsurance [53]. Other studies failed to find an association between employment status and various measures of access [42, 47, 54, 62]. Two studies reported married status was associated with increased health insurance coverage [53, 58], checkups in the last year [58], and decreased avoidance of seeking healthcare because of cost [58] compared to non-married status post-Medicaid expansion. Other studies found no association between marital status and measures of access [42, 47, 54, 58, 62]. Low-income reproductive-age women without dependent children were more likely to have health insurance coverage [52, 58], have a personal doctor [52], have a check-up in the last year [58], and less likely to avoid seeking care because of cost compared to those with dependent children post-Medicaid expansion [58].

Limited evidence was available regarding associations between other individual predisposing factors, including health literacy [42], immigrant status [42, 48, 62], sexual orientation/gender identity status [45, 66], discrimination [66]; English proficiency [62], information sources [42], or family size and the number of births [53, 54] and various measures of access. Higher levels of health literacy were associated with significantly increased odds of having a regular healthcare provider [42]. Immigrants were less likely to have a regular healthcare provider compared to those born in the U.S. [42] or to have visited a doctor at least once in the past year [62]. None of the included studies examined associations between health beliefs, occupation, religion, or social networks and measures of access.

#### Individual enabling factors

Most studies found insurance coverage was associated with higher rates of healthcare utilization, such as visiting a doctor in the last year [54, 62], or receipt of a blood pressure check or influenza vaccination in the last year [60]. Women without insurance or Medicaid/Medi-Cal coverage were less likely to have a regular healthcare provider [42]. We found mixed evidence of an association between income and different access measures. One study found that uninsurance in low-income women declined significantly post-Medicaid expansion, primarily due to increased Medicaid coverage [53]. Another study reported that women in lower income groups (≤138% and 139% −399% FPL) reported improved health insurance affordability and increased doctor’s visits in the past 12 months, and receipt of preventive services increased across all income groups [56]. Other studies found no association between income levels and different measures of access [48, 54, 62, 66]. In reproductive-aged women, higher income levels were associated with increased utilization of preventive health services such as blood pressure checks or influenza vaccinations. There was limited evidence assessing associations between a usual source of care [62], access to public transit [42], geographic residence [54], and measures of access. Women with a usual source of care were more likely to have seen a physician at least once in the past year [62].

#### Individual need factors

The few studies that explored associations between need factors and measures of access found mixed evidence [42, 51, 54, 59, 62]. Women with chronic diseases were more likely to have seen a doctor in the past year [62], and women with hypertension had significantly higher odds of having a regular healthcare provider [42]. Women with mental health problems were less likely to have had a routine health check-up in the last two years (79.5% versus 82.2%) [59]. Evidence was mixed regarding associations between perceived health status and measures of access [42, 54, 62]. Only one study assessed individual health behaviors (tobacco use) and measures of access [42] and found no association.

#### Contextual level factors

None of the included studies examined contextual predisposing factors such as community levels of education, employment, or crime rates or contextual need factors such as environmental health-related measures such as air, housing, or water quality, death, or injury rates with measures of access. One study found that while rural areas had lower per-capital county supplies of nurse practitioners/physician assistants compared to urban areas, urban-rural differences were not significant for per-capital county supplies of family medicine physicians [54].

Several studies examined contextual enabling factors such as ACA’s healthcare policy enacted into law in 2010. Medicaid expansion in designated states was associated with decreased uninsured rates in adult women [55]; women of reproductive age [46, 53], low-income women of reproductive age [43, 52], low-income childless women [64], low-income women [65] and low‐educated adults [67], compared to non-expansion states. For example, the odds of being uninsured were two times higher for women of reproductive age residing in Medicaid non-expansion states compared to expansion states [53]. Increased levels of overall insurance coverage were associated with Medicaid expansion in adult women [44], women of reproductive age [46], low-income childless women [64], and low‐educated women compared to non-expansion states. In several studies, Medicaid expansion was associated with significantly increased rates of coverage in working-age women [43, 44, 46, 55, 64, 67]. One study of low-income reproductive-age women found 38.7% of uninsured women in 2013 acquired Medicaid in 2014 in expansion states compared to 19.5% in non-expansion states [43].

There was mixed evidence of an association between Medicaid expansion and other measures of potential access. Two studies found that Medicaid expansion was significantly associated with increased access to a usual source of care in reproductive-age women [46] and access to a personal doctor in low-income women [65]. A California-based study of low-income reproductive-age women, found those insured with Medi-Cal, other public insurance, or private insurance, compared to women with no insurance, were significantly more likely to have a usual source of care post-expansion [48]. In contrast, other studies found no association between Medicaid expansion and improved access to a usual source of care in adult women [55] or low-income women [49]; having a personal doctor [52, 63], or needing to see a doctor but unable to because of cost [52] in low-income women.

Several studies found Medicaid expansion led to a reduction in delayed or non-receipt of medical care in the past year for both adult women [55] and reproductive-age women [46], the increased ability of low-income women to afford healthcare [65], decreased problems paying medical bills for adult women [55], increased ability to see a doctor due to lower cost for low-income reproductive-age women [52] and decreased avoidance of healthcare due to cost for low-income reproductive-age women [58].

Few included studies examined the impact of Medicaid expansion on women’s healthcare utilization. Medicaid expansion was associated with increased doctor’s visits in women in lower-income brackets (≤138% and 139% −399% FPL), preventive health screenings in all adult women [56], and increased checkup visits in low-income reproductive-age women [58]. However, the majority of studies found no association between Medicaid expansion and provider visits [49, 52], cholesterol checks [58], routine check-ups [63], or a flu shot [63] in the past year in low-income women.

### Overall synthesis of qualitative evidence

A thematic analysis of three qualitative studies examining the experiences of Latina immigrants [50, 57] and Iraqi refugee women [61] was conducted to identify facilitators and barriers to primary care access. Two thematic areas of interest included 1) facilitators and 2) barriers to healthcare access. Themes were grouped according to domains from the Andersen model [31].

#### Facilitators

Five themes were developed relating to facilitators. Three themes aligned with individual predisposing characteristics included 1) positive health beliefs, 2) health-affirming behaviors, and 3) social support. Belief in the efficacy of biomedical options resulted in timely healthcare-seeking behaviors [61]. Women were often motivated to seek healthcare because they wanted to stay healthy so they could take care of their families [57]. Health-affirming behaviors included recognizing the importance of health, applying relevant health-related knowledge, health literacy, and knowledge about community resources such as where to access free, low-cost healthcare [50]. Social support such as family members acting as translators at doctor’s visits or access to social networks including faith communities [57], religious beliefs, and informal networks that assisted women in navigating difficult life circumstances [50] were important coping mechanisms that often facilitated access to care.

Two themes were linked to individual and contextual enabling factors, including 1) healthcare safety net and 2) healthcare organization and delivery. Access to insurance facilitated healthcare access [50]. Often low-income immigrant or refugee women did not have insurance coverage, so accessed free or low-cost healthcare or prescription medications [57]. Healthcare organization characteristics such as close geographic proximity, culturally appropriate healthcare, and the provision of translation services facilitated access to healthcare [50, 57].

#### Barriers

Six themes related to barriers to access included: 1) immigrant status and linguistic barriers, 2) negative health beliefs, 3) inadequate healthcare safety net, 4) healthcare organization and delivery barriers, 5) delayed care, and 6) health service alternatives. Two themes, 1) immigrant status and linguistic barriers, and 2) negative health beliefs related to individual predisposing characteristics. Immigrant identity was linked to low-income status and a lack of English proficiency. Undocumented status was a significant barrier as these women often did not qualify for medical assistance or insurance coverage under federally funded Medicaid or Medicare programs, so could not afford care [50, 57]. Negative health beliefs, such as the perception of health as the absence of illness, also led to delayed health-seeking for preventive care services [50].

The two themes of inadequate healthcare safety net and healthcare organization and delivery barriers were linked to individual and contextual enabling characteristics. Lack of insurance and the high cost of healthcare services are significant barriers to access [50, 57, 61]. Often women are unable to enter the health insurance market if they are not eligible for Medicaid or other publicly funded health insurance and rely on free or low-cost clinics, same-day appointments at urgent care, or a visit to the emergency room as a last resort [57, 61]. Healthcare delivery system-related barriers such as discriminatory practices, difficulties making appointments, inadequate provider assessments or treatments, inexperienced providers, lack of follow-up regarding test results, lack of translators, language difficulties, unreliable public transportation, and long wait times often prevented access to or delivery of effective healthcare services [50, 57, 61].

Finally, two themes delayed care and health service alternatives related to Andersen’s domain of health behaviors. Women often delayed care because of financial constraints and fears about financial costs or competing needs (such as family, and work commitments) or a history of negative experiences with healthcare providers, such as not being listened to [61]. Immigrants felt undeserving of government assistance programs available to U.S. citizens [57]. Shopping around for health services or prescription drugs that were free or low-cost to avoid having to use the emergency room was common [57], which often delayed care or resulted in the receipt of low-quality or inappropriate care. These themes are outlined in the S3 Table with illustrative quotes or excerpts.

### Methodological limitations of studies

Some of the quantitative studies used cross-sectional data so could not assess temporality, or whether causal relationships existed between variables of interest and outcome measures [42, 45, 47–49, 51, 53, 59, 60, 62, 66]. Many of the quantitative studies used secondary datasets from large national surveys such as the BRFSS, NHIS, or MEPS [43–46, 51, 52, 54–56, 58–60, 63, 64, 67], which often include standard self-reported healthcare survey variables, which may limit the applicability of findings to the review questions. Studies conducting secondary data analyses of cross-sectional surveys such as the MEPS and NHIS have higher response rates than the BRFSS [68]. The BRFSS uses random-digit dialed surveys, which may lower individual response rates and frame non-coverage, reducing representativeness [69].

While quantitative studies generally controlled for confounding variables, one study may have failed to control for all potential confounders [60]. In addition, considerable heterogeneity regarding the operationalization of access measures made it difficult to compare the findings of quantitative studies included in the review. For example, definitions of measures of affordability or costs often varied considerably across studies [45, 46, 51, 52, 55, 56, 58, 63, 65]. Additionally, comparison across studies was complicated by the fact that some studies examined associations between measures of potential access [42, 43, 49, 53, 58, 66] or measures of realized access [47, 49, 54, 58, 62] and variables such as education, employment, or marital status. Several studies employed difference-in-difference study designs to examine the effects of ACA [43, 44, 52, 55, 56, 58, 63–65, 67]; however, because of confounding from contemporaneous socio-economic or political changes, these estimates were unable to determine causality. Some difference-in-differences studies used logistic regression models, while others used linear regression models, which may make it easier to interpret the findings of difference-in-difference studies [70].

## Discussion

This systematic review advances our understanding by synthesizing evidence about major determinants, facilitators, and barriers that impact working-age women’s access to primary care in the ACA era. Current gaps in knowledge about this topic are identified. Regarding the first objective, the review found moderate evidence that individual predisposing factors such as age, marital status, race/ethnicity, and enabling factors such as insurance and income were associated with various access measures in working-aged women. Evidence of an association between other individual predisposing, enabling, and need factors and measures of access was often mixed or not reported. Contextual enabling factors such as ACA’s Medicaid expansion were consistently associated with reduced uninsured rates and gains in coverage, particularly Medicaid, among women in low and moderate-income brackets. Evidence of association between Medicaid expansion and other measures of access, such as a source of usual care or regular provider or health service use was mixed.

Regarding the second objective, limited qualitative evidence from studies of low-income immigrant and refugee women suggests that facilitators of access include free or low-cost healthcare, health literacy, social support, and supportive relationships with providers. Barriers include lack of insurance coverage, high costs of health care, healthcare delivery system barriers such as lack of translation services, poor communications with providers, and transportation barriers. The synthesis of evidence in this review builds on findings from other studies that have examined the association of various socio-demographic characteristics with access measures in non-elderly U.S. adults.

The ACA led to some of the most significant advances in women’s access to primary care in recent decades. ACA provisions such as Medicaid expansion resulted in significant decreases in rates of uninsurance and gains in Medicaid coverage for women living in Medicaid expansion states, compared to non-expansion states. Despite these gains, some studies reported differential patterns in accessing health coverage by age, income, marital status, and race/ethnicity persist for women post-Medicaid expansion [53, 55, 58, 67, 71]. Similarly, previous studies of U.S.-based adults have found that while Medicaid expansion reduced inequalities in insurance coverage by age, income level, marital status, and race and ethnicity, substantial inequalities continue to persist among low-income minority adults [72–77]. The findings of our review highlight the need for additional research to identify how differential patterns of access intersect with gender and other determinants, such as age, income, marital status, and race/ethnicity in the ACA era. Research needs to focus on low-income women or women belonging to racial/ethnic or sexual minority groups who are most often impacted by healthcare inequalities.

In this review, we found few studies had assessed the effect of ACA provisions, such as Medicaid expansion on women’s coverage, access, and healthcare utilization [43, 44, 46, 48, 49, 52, 53, 55, 56, 58, 63–65, 67]. A recent literature review of 601 studies published between 2014 to 2021 examined the effect of Medicaid expansion on outcomes relating to access and coverage, affordability, and healthcare service use for working-age adults [78, 79]. This literature review revealed few studies have examined how the ACA has impacted working-age women’s access to care, and often studies failed to report subgroup gender analyzes [75, 80–83]. Studies about women’s access included in the review often focused on the impact of ACA provisions on access to reproductive care [3, 84–91].

Insurance coverage is the most commonly reported indicator of access in ACA-related literature, however, may not always lead to improvement in realized measures of access. There may be several reasons ACA provisions are not always associated with improved access, affordability, or health service use for women. A nationally representative survey conducted in 2022 with working-age women found that 68% of low-income women and 52% of all participating women experienced difficulties paying for medical bills and also found it difficult to pay for necessities like food, heat, or housing [6]. Under ACA provisions, insurance coverage may not sufficiently protect against high costs, or networks may be so limited that newly insured individuals cannot find care [92]. Women, especially if uninsured, underinsured, or low-income, may be more likely to delay or forego care if unable to afford health care costs or medical bills.

Low-income women with Medicaid coverage are more likely to report health insurance coverage with limitations compared to those with private insurance (individual or employer-sponsored) [6]. Women may also be subject to coverage exclusions for certain types of conditions, health maintenance services, or preventive services not mandated by law [93]. There is a need for quantitative research that evaluates the longer-term impact of the ACA on women’s access, coverage, and use of primary care services. Highlighted research gaps include the effect of certain ACA provisions such as Marketplace subsidies or the Individual Mandate on coverage, affordability, and utilization for women [30].

In the context of the ACA, limited qualitative or mixed-methods research has also been conducted on women’s experiences accessing primary care, factors that act as facilitators or barriers to access, satisfaction with care received, and health outcomes. We found limited qualitative research on women’s experiences of accessing primary care. Qualitative studies included in this review focused on immigrant and refugee women’s experiences [50, 57, 61]. In the ACA era, qualitative research has often focused on facilitators and barriers to access in broad adult populations [94–98]. Other studies of immigrant and refugee women since ACA have focused on specific sub-groups such as East African-born women (18–85 years) [99], undocumented African immigrant women’s experiences with ambulatory care visits, emergency room and hospital admission [100], homeless women [101], and women receiving reproductive health services [102–105].

Our findings are consistent with those of other U.S.-based qualitative studies of working-age women and their experiences accessing cancer screening or sexual and reproductive health services in the ACA era. Several studies reported facilitators of access included health coverage, good health literacy [99, 105, 106], social support [102, 104, 106], and positive interactions with providers [103, 105]; while common barriers included no health coverage or high costs [99, 102, 104, 107], low health literacy [99, 104–106] healthcare delivery barriers such as inadequate provider communication or mistrust [99, 102, 105], lack of culturally appropriate services [99, 107], and transportation issues [102, 104, 105, 107]. Several of these studies also found that difficulties navigating providers or clinics were a significant barrier [102, 104]. Similarly, an Oregon-based qualitative study found new Medicaid adult enrollees often failed to access care regularly because of barriers, including poor interactions with providers, confusion about coverage, or a lack of perception about the need for medical care [94]. Myriad other facilitators and barriers may impact working-age women’s access to primary care in the ACA era and warrant further investigation.

This review identified there is a need for additional research to explore the experiences of broader heterogeneous populations of women, to ascertain the complex factors that deter working-age women’s access to primary care despite ACA provisions that have expanded coverage. Quantitative studies rarely considered individual predisposing or enabling factors, such as health literacy, information sources, language barriers, social support networks, or means of transportation. Qualitative or mixed methods research focusing on vulnerable groups of women can provide valuable insights to inform health-related policies, programs, and interventions aimed at improving women’s access to primary care in U.S.-based settings and beyond.

### Implications

#### Health policy initiatives

To address contextual enabling factors such as lack of insurance coverage, expanding Medicaid eligibility to the remaining ten states that have not adopted Medicaid expansion would provide essential insurance coverage to eligible low-income women. There is a need to increase federal and state outreach to uninsured women who are eligible but not yet enrolled in Medicaid or marketplace coverage [4, 108]. The American Rescue Plan (ARP) of 2021 has enabled many Americans to access affordable coverage, qualifies those previously eligible for Premium Tax Credits for increased subsidies, and reduces premiums [109]. Under the ARP, approximately 2.6 million uninsured Hispanics may qualify for zero-dollar insurance plans, and an additional 3 million may qualify for low-cost premiums [110]. Continued support for the ARP will help to address healthcare coverage inequities, especially in uninsured or underinsured Hispanic populations. National reform of immigration policies with provisions that reduce restrictions for undocumented immigrant eligibility for Medicaid and marketplace insurance plans would help reduce the much higher rates of uninsurance in Latinas [111].

Other measures such as redesigning eligibility requirements for cost-sharing and premium tax credits to promote affordable insurance for individuals, instituting federal and state regulations limiting the availability of individual market plans that are non-compliant with ACA regulation, funding programs offering consumer assistance, education, and outreach promoting open enrolment, and general assistance with enrolment, and federal legislation authorizing a public insurance plan that provides various options for coverage as a stepping stone to universal coverage should be considered [108].

#### Navigating healthcare access

Many women without coverage would benefit from assistance with navigating access to insurance coverage. In 2021, the Centers for Medicare and Medicaid Services announced it would provide $80 million to fund navigators ahead of and during 2022 open enrollment for health insurance to support educational outreach and assistance with navigation, with a focus on culturally responsive strategies to redress budget cuts to the navigator program beginning in 2019 [112]. More culturally tailored navigation programs initiated by federal, state and local governments could help to identify and assist low-income or otherwise vulnerable groups of women who are eligible for Medicaid, tax credits, or marketplace schemes with signing up for Medicaid coverage or other provisions of the ACA.

#### Delivery of culturally appropriate care

To promote healthcare access, individual-level interventions that provide more culturally appropriate care for immigrant groups of women including linguistic and/or cultural matching of providers with patients, incorporation of culturally specific components within individual encounters, provision of culturally/linguistic appropriate materials, family involvement, and continuity of care are needed [113]. Healthcare delivery organizations can provide training in cultural competency, integrate interpreter services into the delivery of services, employ community health workers to provide culturally appropriate care, provide telemedicine services, and conduct outreach [113].

### Strengths and limitations

This review provides insights into multitudinous factors that can impact women’s access to primary care during the ACA and provides suggestions for future research, policy, and practice. Adopting a mixed-methods approach allowed for the inclusion of heterogeneous study designs, analytic methods, measures, and outcomes to meet the broad and diverse research questions. Applying Anderson’s model as a framework to examine determinants and factors associated with access may contribute to the generalizability of findings to women living in other settings. While this review only includes U.S.-based studies, these findings may be relevant to other countries, especially high or middle-income countries, who have not adopted universal healthcare coverage and have significant uninsured or underinsured populations. Despite a systematic search of several databases, as the research questions were broad, selective use of MeSH headings and key terms and exclusion of non-English studies may have excluded potentially eligible studies. Despite this limitation, it is unlikely that the review findings would be significantly altered as key relevant studies conducted with women were included.

### Future directions

This review provides additional insights into factors that may impact women’s access to health care during the ACA and provides suggestions for future research, policy, and practice. This review is relevant to policymakers, healthcare administrators, public health professionals, and healthcare providers in the U.S. and other countries experiencing similar challenges to healthcare access because of a lack of universal healthcare coverage. More longitudinal studies using time-series or repeated measures analysis would be helpful to explore trends relating to individual or social determinants of health such as available types of insurance coverage, to capture any changes that have occurred following the implementation of major ACA provisions relating to women’s access to healthcare including reproductive services. Strategies to improve women’s access to care should first focus on assisting women who may be eligible under ACA provisions to obtain health coverage and developing tailored, culturally sensitive programs. Second, more innovative interventions and strategies need to be developed to assist women in navigating access to health insurance and promoting women’s regular engagement with primary care services for acute, chronic and preventive healthcare needs.

Further work needs to be done to promote healthcare-related immigration policies at the federal, state, and local levels to address inequities around access in vulnerable, often marginalized immigrant and refugee groups of women. The engagement of federal and state-level health policymakers in developing relevant policies and federal and state funding is essential for any necessary reforms. Other stakeholders, including public health professionals, frontline providers such as primary care providers and nurses, and women, should be included in the planning design, and implementation of programs and interventions to improve women’s access to primary care. Policymakers, healthcare system administrators, researchers, and healthcare providers must work together to fund, design, and develop equitable healthcare policies, programs, and interventions aimed at improving healthcare access for all working-age women.

## Supporting information

S1 File PRISMA 2020 checklist. (PDF)

S2 File Database search strategies. (PDF)

S3 File Data extraction form. (XLSX)

S4 File Quality appraisal of studies applying Mixed Methods Appraisal Tool (MMAT) version 2018. (PDF)

S1 Table Inclusion and exclusion criteria. (PDF)

S2 Table Determinants of access and utilization of healthcare services for working-age women. (PDF)

S3 Table Andersen’s model domains and themes relating to facilitators and barriers to access. (PDF)
