# Supplementary material for: Factors affecting women’s access to primary care in the United States since the Affordable Care Act: A mixed-methods systematic review
Source: PLoS One. 2024 Dec 19;19(12):e0314620. doi: 10.1371/journal.pone.0314620 (PMC11658587; doi:10.1371/journal.pone.0314620)
Supplement: S2 File — (PDF) [file pone.0314620.s002.pdf]

S2 File. Database search strategies.

MEDLINE search strategy

#	Query	Limiters/Expanders	Last Run Via
S15	S11 AND S14	Limiters - Date of Publication: 20100101-; Abstract Available; English Language; Human; Age Related: All Adult: 19+ years; Scholarly (Peer Reviewed) Journals Search modes - Find all my search terms	Interface - EBSCOhost Research Databases Search Screen - Advanced Search Database - MEDLINE Complete
S14	S12 OR S13	Search modes - Find all my search terms	Interface - EBSCOhost Research Databases Search Screen - Advanced Search Database - MEDLINE Complete
S13	AB (woman* OR women* OR female* OR gender* OR mother*) OR TI (woman* OR women* OR female* OR gender* OR mother*)	Search modes - Find all my search terms	Interface - EBSCOhost Research Databases Search Screen - Advanced Search Database - MEDLINE Complete
S12	(MH "Women+") OR (MH "Female") OR (MH "Gender Identity+")	Search modes - Find all my search terms	Interface - EBSCOhost Research Databases Search Screen - Advanced Search Database - MEDLINE Complete
S11	S7 AND S10	Search modes - Find all my search terms	Interface - EBSCOhost Research Databases Search Screen - Advanced Search Database - MEDLINE Complete
S10	S8 OR S9	Search modes - Find all my search terms	Interface - EBSCOhost Research Databases Search Screen - Advanced Search Database - MEDLINE Complete
S9	AB ("united states" OR america OR usa OR u.s.a OR u.s OR "united states of america") OR TI ("united states" OR america OR usa OR u.s.a OR u.s OR "united states of america")	Search modes - Find all my search terms	Interface - EBSCOhost Research Databases Search Screen - Advanced Search Database - MEDLINE Complete
S8	(MH "United States+")	Search modes - Find all my search terms	Interface - EBSCOhost Research Databases Search Screen - Advanced Search Database - MEDLINE Complete
S7	S3 AND S6	Search modes - Find all my search terms	Interface - EBSCOhost Research Databases Search Screen - Advanced Search Database - MEDLINE Complete
S6	S4 OR S5	Search modes - Find all my search terms	Interface - EBSCOhost Research Databases Search Screen - Advanced Search

#	Query	Limiters/Expanders	Last Run Via
			Database - MEDLINE Complete
S5	AB ((access* OR avail* OR use OR usage OR acceptance OR utili*) N5 (healthcare OR "health care" OR health-care) OR TI ((access* OR avail* OR use OR usage OR acceptance OR utili*) N5 (healthcare OR "health care" OR health-care)	Search modes - Find all my search terms	Interface - EBSCOhost Research Databases Search Screen - Advanced Search Database - MEDLINE Complete
S4	(MH "Health Services Accessibility+") OR (MH "Health Services Needs and Demand+")	Search modes - Find all my search terms	Interface - EBSCOhost Research Databases Search Screen - Advanced Search Database - MEDLINE Complete
S3	S1 OR S2	Search modes - Find all my search terms	Interface - EBSCOhost Research Databases Search Screen - Advanced Search Database - MEDLINE Complete
S2	AB ((primary OR preventative OR preventive) N5 (care OR "health care" OR healthcare OR medicine OR service*)) OR TI ((primary OR preventative OR preventive) N5 (care OR "health care" OR healthcare OR medicine OR service*))	Search modes - Find all my search terms	Interface - EBSCOhost Research Databases Search Screen - Advanced Search Database - MEDLINE Complete
S1	(MH "Primary Health Care+") OR (MH "Preventive Health Services+") OR (MH "Preventive Medicine+") OR (MH "Women's Health Services+")	Search modes - Find all my search terms	Interface - EBSCOhost Research Databases Search Screen - Advanced Search Database - MEDLINE Complete

CINAHL search strategy

#	Query	Limiters/Expanders	Last Run Via
S16	S11 AND S14	Limiters - Abstract Available; Published Date: 20100101-; English Language; Peer Reviewed; Human; Age Groups: All Adult Search modes - Find all my search terms	Interface - EBSCOhost Research Databases Search Screen - Advanced Search Database - CINAHL
S15	S11 AND S14	Search modes - Find all my search terms	Interface - EBSCOhost Research Databases Search Screen - Advanced Search Database - CINAHL
S14	S12 OR S13	Search modes - Find all my search terms	Interface - EBSCOhost Research Databases Search Screen - Advanced Search Database - CINAHL

#	Query	Limiters/Expanders	Last Run Via
S13	AB ((woman* OR women* OR female* OR gender* OR mother*) OR TI (woman* OR women* OR female* OR gender* OR mother*))	Search modes - Find all my search terms	Interface - EBSCOhost Research Databases Search Screen - Advanced Search Database - CINAHL
S12	(MH "Female") OR (MH "Women+") OR (MH "Gender Identity+")	Search modes - Find all my search terms	Interface - EBSCOhost Research Databases Search Screen - Advanced Search Database - CINAHL
S11	S7 AND S10	Search modes - Find all my search terms	Interface - EBSCOhost Research Databases Search Screen - Advanced Search Database - CINAHL
S10	S8 OR S9	Search modes - Find all my search terms	Interface - EBSCOhost Research Databases Search Screen - Advanced Search Database - CINAHL
S9	AB (("united states" OR america OR usa OR u.s.a OR u.s or "united states of america") OR TI ("united states" OR america OR usa OR u.s.a OR u.s or "united states of america"))	Search modes - Find all my search terms	Interface - EBSCOhost Research Databases Search Screen - Advanced Search Database - CINAHL
S8	(MH "United States+")	Search modes - Find all my search terms	Interface - EBSCOhost Research Databases Search Screen - Advanced Search Database - CINAHL
S7	S3 AND S6	Search modes - Find all my search terms	Interface - EBSCOhost Research Databases Search Screen - Advanced Search Database - CINAHL
S6	S4 OR S5	Search modes - Find all my search terms	Interface - EBSCOhost Research Databases Search Screen - Advanced Search Database - CINAHL
S5	AB ((access* OR avail* OR use OR usage OR acceptance OR utili*) N5 (healthcare OR "health care" OR health-care)) OR TI ((access* OR avail* OR use OR usage OR acceptance OR utili*) N5 (healthcare OR "health care" OR health-care))	Search modes - Find all my search terms	Interface - EBSCOhost Research Databases Search Screen - Advanced Search Database - CINAHL
S4	(MH "Health Services Accessibility+") OR (MH "Health Services Needs and Demand+")	Search modes - Find all my search terms	Interface - EBSCOhost Research Databases Search Screen - Advanced Search Database - CINAHL
S3	S1 OR S2	Search modes - Find all my search terms	Interface - EBSCOhost Research Databases Search Screen - Advanced Search Database - CINAHL
S2	AB ((primary OR preventative OR preventive) N5 (care OR	Search modes - Find all my search terms	Interface - EBSCOhost Research Databases Search

#	Query	Limiters/Expanders	Last Run Via
	"health care" OR healthcare OR medicine OR service*)) OR TI ((primary OR preventative OR preventive) N5 (care OR "health care" OR healthcare OR medicine OR service*))		Screen - Advanced Search Database - CINAHL
S1	(MH "Primary Health Care") OR (MH	Search modes - Find all my search terms	Interface - EBSCOhost Research Databases Search Screen - Advanced Search Database - CINAHL

PsycINFO search strategy

#	Query	Limiters/Expanders	Last Run Via
S14	S9 AND S12	Limiters - Published Date: 20100101-; Peer Reviewed; English; Age Groups: Adulthood (18 yrs & older); Population Group: Human Search modes - Find all my search terms	Interface - EBSCOhost Research Databases Search Screen - Advanced Search Database - APA PsycInfo
S13	S9 AND S12	Search modes - Find all my search terms	Interface - EBSCOhost Research Databases Search Screen - Advanced Search Database - APA PsycInfo
S12	S10 OR S11	Search modes - Find all my search terms	Interface - EBSCOhost Research Databases Search Screen - Advanced Search Database - APA PsycInfo
S11	AB ((woman* OR women* OR female* OR gender* OR mother*)) OR TI ((woman* OR women* OR female* OR gender* OR mother*))	Search modes - Find all my search terms	Interface - EBSCOhost Research Databases Search Screen - Advanced Search Database - APA PsycInfo
S10	(DE "Human Females" OR DE "Battered Females" OR DE "Daughters" OR DE "Female Criminal Offenders" OR DE "Mothers" OR DE "Sisters" OR DE "Widows" OR DE "Wives" OR DE "Working Women") OR (DE "Gender Identity" OR DE "Gender Nonconforming" OR DE "LGBTQ" OR DE "Transgender" OR DE "Transsexualism")	Search modes - Find all my search terms	Interface - EBSCOhost Research Databases Search Screen - Advanced Search Database - APA PsycInfo
S9	S7 AND S8	Search modes - Find all my search terms	Interface - EBSCOhost Research Databases Search Screen - Advanced Search Database - APA PsycInfo
S8	AB ("united states" OR america OR usa OR u.s.a OR u.s OR "united states of america") OR TI ("united states" OR america OR usa	Search modes - Find all my search terms	Interface - EBSCOhost Research Databases Search Screen - Advanced Search Database - APA PsycInfo

#	Query	Limiters/Expanders	Last Run Via
	OR u.s.a OR u.s OR "united states of america")		
S7	S3 AND S6	Search modes - Find all my search terms	Interface - EBSCOhost Research Databases Search Screen - Advanced Search Database - APA PsycInfo
S6	S4 OR S5	Search modes - Find all my search terms	Interface - EBSCOhost Research Databases Search Screen - Advanced Search Database - APA PsycInfo
S5	AB ((access* OR avail* OR use OR usage OR acceptance OR utili*) N5 (healthcare OR "health care" OR health-care)) OR TI ((access* OR avail* OR use OR usage OR acceptance OR utili*) N5 (healthcare OR "health care" OR health-care))	Search modes - Find all my search terms	Interface - EBSCOhost Research Databases Search Screen - Advanced Search Database - APA PsycInfo
S4	(DE "Health Care Utilization" OR DE "Utilization Reviews" OR DE "Health Care Access" OR DE "Treatment Barriers" OR DE "Health Care Seeking Behavior" OR DE "Professional Referral" OR DE "Self- Referral" OR DE "Health Service Needs")	Search modes - Find all my search terms	Interface - EBSCOhost Research Databases Search Screen - Advanced Search Database - APA PsycInfo
S3	S1 OR S2	Search modes - Find all my search terms	Interface - EBSCOhost Research Databases Search Screen - Advanced Search Database - APA PsycInfo
S2	AB ((primary OR preventative OR preventive) N5 (care OR "health care" OR healthcare OR medicine OR service*)) OR TI ((primary OR preventative OR preventive) N5 (care OR "health care" OR healthcare OR medicine OR service*))	Search modes - Find all my search terms	Interface - EBSCOhost Research Databases Search Screen - Advanced Search Database - APA PsycInfo
S1	(DE "Primary Health Care") OR (DE "Preventive Health Services" OR DE "Prophylactic Drug Therapy")	Search modes - Find all my search terms	Interface - EBSCOhost Research Databases Search Screen - Advanced Search Database - APA PsycInfo

Web of Science search strategy

#	Query
S13	(#10 AND #9) AND LANGUAGE: (English)<br>Refined by: PUBLICATION YEARS: (2021 OR 2013 OR 2020 OR 2012 OR 2019 OR 2011 OR 2018 OR 2010 OR 2017 OR 2016 OR 2015 OR 2014)<br>Indexes=SCI-EXPANDED, SSCI Timespan=1900-2021
S12	(#10 AND #9) AND LANGUAGE: (English)<br>Indexes=SCI-EXPANDED, SSCI Timespan=1900-2021
S11	#10 AND #9<br>Indexes=SCI-EXPANDED, SSCI Timespan=1900-2021
S10	TS=(woman* OR women* OR female* OR gender* OR mother*)<br>Indexes=SCI-EXPANDED, SSCI Timespan=1900-2021
S9	#8 AND #7<br>Indexes=SCI-EXPANDED, SSCI Timespan=1900-2021
S8	TS=("united states" OR america OR usa OR u.s.a OR u.s OR "united states of america")<br>Indexes=SCI-EXPANDED, SSCI Timespan=1900-2021
S7	#6 AND #3<br>Indexes=SCI-EXPANDED, SSCI Timespan=1900-2021
S6	#5 OR #4<br>Indexes=SCI-EXPANDED, SSCI Timespan=1900-2021
S5	AB=((access* OR avail* OR use OR usage OR acceptance OR utili*) NEAR/5 (healthcare OR "health care" OR health-care)) OR TI=((access* OR avail* OR use OR usage OR acceptance OR utili*) NEAR/5 (healthcare OR "health care" OR health-care))<br>Indexes=SCI-EXPANDED, SSCI Timespan=1900-2021
S4	TS=("health service access" OR "health service need" OR "health service demand" OR "health service utilization" OR "health service utilisation" OR "health need" OR "health care need" OR "healthcare need" OR "health care demand" OR "healthcare demand")<br>Indexes=SCI-EXPANDED, SSCI Timespan=1900-2021
S3	#2 OR #1<br>Indexes=SCI-EXPANDED, SSCI Timespan=1900-2021
S2	AB=((primary OR preventative OR preventive) NEAR/5 (care OR "health care" OR healthcare OR medicine OR service*)) OR TI=((primary OR preventative OR preventive) NEAR/5 (care OR "health care" OR healthcare OR medicine OR service*))<br>Indexes=SCI-EXPANDED, SSCI Timespan=1900-2021
S1	TS=("primary care" OR "primary health care" OR "preventive medicine" OR "preventive services" OR "preventive health services" OR "women's health" OR "womens health")<br>Indexes=SCI-EXPANDED, SSCI Timespan=1900-2021

Note

Database searches of MEDLINE, CINAHL, PsycINFO, and Web of Science were conducted on 25 and 26 January 2021. An updated search in MEDLINE, CINAHL, PsycINFO and Web of Science using the same search strategies was conducted on 10 October 2023.
