# Supplementary material for: Factors affecting women’s access to primary care in the United States since the Affordable Care Act: A mixed-methods systematic review
Source: PLoS One. 2024 Dec 19;19(12):e0314620. doi: 10.1371/journal.pone.0314620 (PMC11658587; doi:10.1371/journal.pone.0314620)
Supplement: S4 File — (PDF) [file pone.0314620.s004.pdf]

S4 File. Quality appraisal of studies applying Mixed Methods Appraisal Tool (MMAT) Version 2018.

		SCREENING QUESTIONS		1. QUALITATIVE STUDIES					3. NON-RANDOMIZED STUDIES					
**First author**	**Year**	**S1.**	**S2.**	**1.1.**	**1.2.**	**1.3.**	**1.4. Is**	**1.5.**	**3.1.**	**3.2.**	**3.3.**	**3.4.**	**3.5.**	**Score**
Ahad	2019	Yes	Yes						No	Yes	Yes	Yes	Yes	4/5, 80%
Chen	2020	Yes	Yes						Can’t tell	Yes	Yes	Yes	Yes	4/5, 80%
Courte-manche	2019	Yes	Yes						Yes	Yes	Can't tell	Yes	Yes	4/5, 80%
Dai	2019	Yes	Yes						Yes	Yes	Yes	Yes	Yes	5/5, 100%
Daw	2019	Yes	Yes						Yes	Yes	Yes	Yes	Yes	5/5, 100%
Early	2018	Yes	Yes						Yes	Yes	Can't tell	Yes	Yes	4/5, 80%
Farietta	2018	Yes	Yes						Yes	Yes	Yes	Yes	Yes	5/5, 100%
Greder	2019	Yes	Yes	Yes	Yes	Yes	Yes	Yes						5/5, 100%
Johnson	2020	Yes	Yes						Yes	Yes	Yes	Yes	Can't tell	4/5, 80%
Johnston	2019	Yes	Yes						Yes	Yes	Yes	Yes	Yes	5/5, 100%
Jones	2016	Yes	Yes						No	Yes	Yes	Yes	Yes	4/5, 80%
Lee	2020	Yes	Yes						Yes	Yes	Yes	Yes	Yes	5/5, 100%
Lee	2019	Yes	Yes						Yes	Yes	Yes	Yes	Yes	5/5, 100%%
Lee	2019	Yes	Yes						Yes	Yes	No	Yes	Yes	4/5, 80%
Luque	2018	Yes	Yes	Yes	Yes	Yes	Yes	Yes						5/5, 100%
DiPietro Mager	2021	Yes	Yes						No	Yes	Yes	Yes	Can't tell	3/5, 60%
Margeri-son	2020	Yes	Yes						Yes	Yes	Yes	Yes	Yes	5/5, 100%
Massetti	2017	Yes	Yes						Yes	Yes	Yes	Yes	Yes	5/5, 100%

		SCREENING QUESTIONS		1. QUALITATIVE STUDIES					3. NON-RANDOMIZED STUDIES					
**First author**	**Year**	**S1.**	**S2.**	**1.1.**	**1.2.**	**1.3.**	**1.4. Is**	**1.5.**	**3.1.**	**3.2.**	**3.3.**	**3.4.**	**3.5.**	**Score**
Pazol	2018	Yes	Yes						Yes	Yes	Yes	No	Can't tell	3/5, 60%
Ross Perfetti	2019	Yes	Yes	Yes	Yes	Yes	Can't tell	Yes						4/5, 80%
Seo	2019	Yes	Yes						Yes	Yes	Yes	Yes	Yes	5/5, 100%
Simon	2017	Yes	Yes						Yes	Yes	Yes	Yes	Yes	5/5, 100%
Sommers	2014	Yes	Yes						Yes	Yes	Can't tell	Yes	Can't tell	3/5, 60%
Sommers	2015	Yes	Yes						Can't tell	Yes	Yes	Yes	Yes	4/5, 80%
SteelFish-er	2019	Yes	Yes						Yes	Can't tell	Yes	Yes	Can't tell	3/5, 60%
Wehby	2018	Yes	Yes						Yes	Yes	Yes	Yes	Yes	5/5, 100%
