# Supplementary material for: Factors affecting women’s access to primary care in the United States since the Affordable Care Act: A mixed-methods systematic review
Source: PLoS One. 2024 Dec 19;19(12):e0314620. doi: 10.1371/journal.pone.0314620 (PMC11658587; doi:10.1371/journal.pone.0314620)
Supplement: S1 Table — (PDF) [file pone.0314620.s005.pdf]

S1 Table. Inclusion and exclusion criteria.

Inclusion criteria	Exclusion criteria
Population: Adult women of working age (18-64 years)	-Women living in institutional settings (e.g., prisons, shelters, residential care settings, or inpatient settings)<br>-Women receiving care from the Veterans Health Administration, Military Health System facilities, or the Indian Health Service<br>-Women aged 65 years or older with Medicare coverage<br>-Women experiencing homelessness<br>- Specific sub-populations of women impacted by ACA provisions such as the Dependent-Coverage Mandate or ACA provisions targeting pregnant women<br>-Women employed in specific occupations
Intervention: Provision of formal, face-to-face primary health services provided by a non-specialist primary care provider (e.g., physician, Nurse Practitioner, or Physician's Assistant)	-Studies reporting outcome measures for secondary or tertiary health services (e.g., emergency room, urgent care, specialty outpatient services, specialist mental health services, hospital inpatient services)<br>-Studies reporting utilization of online or telephone-based primary care services
**Outcomes**<br>**Measures of Potential Access**<br>-Health insurance<br>-Type of health insurance<br>-Usual source of primary care or whether have a regular primary care provider<br>-Other barriers to access (e.g., cost barriers, avoided, delayed care due to cost or other issues, unmet need)<br><br>**Measures of realized access**<br>-Utilization (e.g., use/non-use as a binary variable or frequency of use) of primary care services (e.g., acute, chronic healthcare)<br>-Preventive health screenings (e.g., annual physical exam, BP check, cholesterol screening, influenza vaccination)	-Studies not reporting outcomes of potential or realized access to primary care services or routine preventive services for women<br>-Studies reporting women's utilization of sexual and reproductive healthcare services (prenatal, peri-natal, post-natal)<br>-Studies reporting utilization of breast, cervical, or colorectal cancer screening services;<br>-Studies reporting utilization of mental health services in specialty settings;<br>-Studies reporting utilization of dental care services;<br>-Studies reporting disease-specific care (such as for cancer, diabetes, heart disease, and mental health disorders)<br>-Studies reporting treatment or adherence, quality of treatment, patient or provider preferences, or patient satisfaction measures in primary care settings

	-Studies reporting health-related interventions implemented to improve access to/or utilization of primary care
Primary peer-reviewed empirical research, including quantitative observational or experimental studies, qualitative studies, and mixed methods studies	-Consensus, health policy documents, or other gray literature reporting non-peer-reviewed empirical research, book chapters, conference abstracts, dissertations, editorial commentary or opinion papers,<br>-Studies that do not disaggregate relevant findings by sex/gender<br>-Quantitative studies that report simple descriptive data without regression analyses,<br>- The most recent publication reporting outcome measures from specific data sources will be reported to avoid repetition of findings.
Studies conducted in the U.S.	All other high-income countries, middle or low-income countries
English, full-text studies	Non-English language, full-text studies not available.
2010 to 2021	Before 2010
