# Supplementary material for: Factors affecting women’s access to primary care in the United States since the Affordable Care Act: A mixed-methods systematic review
Source: PLoS One. 2024 Dec 19;19(12):e0314620. doi: 10.1371/journal.pone.0314620 (PMC11658587; doi:10.1371/journal.pone.0314620)
Supplement: S2 Table — (PDF) [file pone.0314620.s006.pdf]

S2 Table. Determinants of access and utilization of healthcare services for working-age women.

Andersen's domains		Measures of potential access			Measures of realized access				
**Type**	**Determinants/ factors**	**Insurance coverage/type**	**Usual source of care/regular primary care provider**	**Barriers to care, delayed, foregone care, unmet need**	**Utilization (use/non-use or frequency of use)**	**Preventive health visits (annual physical exam/routine checkup in last year)**	**Preventive health screening (blood pressure)**	**Preventive health screening (cholesterol screening)**	**Preventive health screening (Influenza shot)**
**Individual Predisposing factors**	**Age**	**Chen (2020)** **Gained Medicaid Coverage in 2014 in Expansion States (N=149)** **Uninsured in 2013:** NS (18-23 years); NS (24-33 years); [Ref = 34-44 years]. **Jones & Sonfield (2016),** **Post Medicaid Expansion** **Uninsured:** OR = 1.58, 95% (CI) [1.17–2.12], p = 0.003, (25-29 years); OR = 1.54, 95% CI [1.09–2.18], p = 0.014 (30-34 years); OR = 1.47, 95% CI [1.04–2.09], p = 0.031, (35-39 years) (Ref - 18-24 years). **Lee (2018),**	**Ahad (2019),** **Regular healthcare provider:** OR = .35, 95% CI [.14-.89], p < .05 (18-39 years). **Early (2018)** **Usual source of healthcare** **(Adjusted Prevalence Ratios)** NS (26-29); NS (30-34); aPR = 1.10 [95% CI [1.01, 1.21] (35-39); aPR = 1.12 [95% CI [1.02, 1.22] (40-44); (Ref = 18-25). **Early (2018)** **Post-Medicaid Expansion** **Has Usual Source of Care:**	**SteelFisher (2019),** **Avoided doctor due to discrimination concerns:** NS (30-49 years); NS (50-64 years) (Ref = 18-29 years). **Early (2018)** **Able to obtain timely medical care** **(Adjusted Prevalence Ratios)** NS (26-29); NS (30-34); aPR = 0.86 [95% CI [0.78, 0.95] (35-39) aPR = 0.83 [95% CI [0.75, 0.91] (40-44) (Ref = 18-25); **Able to obtain needed prescriptions**	**Lee (2020),** **Family physician visits (past year)**: 0.3%, 95% CI [0.2, 0.4], p < .001; **NP/PA visits (past year):** NS. **Seo (2019), Doctors visit in the past year:** NS (All women, 40–64); NS (native-born, non-Hispanic White American (NBWA) women, 40–64); NS (Foreign-born Asian immigrant (FBAI) women, 40–64) (Ref = 18-39 years).	**DiPietro Mager (2021),** **Routine check-up past year:** NS (age).	**Pazol (2017),** **BP checked by healthcare professional in past year**: 76.3%, 95% CI [69.6–81.9] (18-19 years); 77.5%, 95% CI [74.2–80.5] (20–24 years); 82.0%, 95% CI [80.2–83.6] (25–34 years); 82.9%, 95% CI [81.1–84.5] (35–44 years).		**Pazol (2017),** **Received influenza vaccine in past year**: 25.1 %, 95% CI [19.6–31.4] (18-19 years.); 24.1 %, 95% CI [21.2–27.3] (20–24 years.); 33.0 %, 95% CI [31.1–35.0] (25–34 years.); 35.9%, 95% CI [33.8–38.1] (35–44 years).

Andersen's domains		Measures of potential access			Measures of realized access				
Type	Determinants/ factors	Insurance coverage/type	Usual source of care/regular primary care provider	Barriers to care, delayed, foregone care, unmet need	Utilization (use/non-use or frequency of use)	Preventive health visits (annual physical exam/routine checkup in last year)	Preventive health screening (blood pressure)	Preventive health screening (cholesterol screening)	Preventive health screening (Influenza shot)
		**Post Medicaid Expansion** **(Difference in percentage-point change between FPL groups),** **Uninsured:** **≤ 138% FPL** -13.78%, 95% CI [-15.78, −11.77] (19-34 years); −14.03%, 95% CI [−17.08, −10.98] (35–44 years); -17.68%, 95% CI [−20.53, −14.84] (45–54 years); −12.88%, 95% CI [−15.81, −9.95] (55–64 years). **139–399% FPL** −9.57%, 95% CI [−11.08, −8.06] (19-34 years); −7.16%, 95% CI [−9.04, − 5.29] (35–44 years); −10.06%, 95% CI [−11.61, −8.51] (45–54 years); −7.71%, 95% CI [−9.42, −5.99] (55–64 years). **Difference in percentage-point**	**(Adjusted Prevalence Ratios)** **Age 18-34 years (n=2,895)** NS (2014); NS (2015); NS (2016); (Ref = 2013); **Age 35-44 years (n=1,672)** NS (2014); NS (2015); NS (2016); (Ref = 2013); P for interaction (year*age) = 0.05.	**(Adjusted Prevalence Ratios)** NS (26-29); aPR = 0.92 [95% CI [0.86, 0.99] (30-34); aPR = 0.91 [95% CI [0.84, 0.99] (35-39) aPR = 0.86 [95% CI [0.79, 0.93] (40-44) (Ref = 18-25). **Early (2018)** **Post-Medicaid Expansion** **Able to obtain timely medical care:** **(Adjusted Prevalence Ratios)** **Age 18-34 years (n=2,895)** **NS (2014);** **NS (2015);** **NS (2016);** **(Ref = 2013);** **Age 35-44 years (n=1,672)** **NS (2014);**					

Andersen's domains		Measures of potential access			Measures of realized access				
**Type**	**Determinants/ factors**	**Insurance coverage/type**	**Usual source of care/regular primary care provider**	**Barriers to care, delayed, foregone care, unmet need**	**Utilization (use/non-use or frequency of use)**	**Preventive health visits (annual physical exam/routine checkup in last year)**	**Preventive health screening (blood pressure)**	**Preventive health screening (cholesterol screening)**	**Preventive health screening (Influenza shot)**
		**<u>change between FPL groups</u>**<br>**≤ 138% FPL vs. 139–399% FPL**<br>−4.21%, 95% CI [−6.87, −1.54] (19-34 years);<br>−6.87%, 95% CI [−10.44, −3.30] (35–44 years);<br>−7.62%, 95% CI [−10.95, −4.3] (45–54 years);<br>−5.18%, 95% CI [−8.53, −1.82] (55–64 years).<br>**Wehby (2018),**<br>**<u>Post Medicaid Expansion (2014-2015)</u>**<br>**Medicaid Insurance**:<br>6.9%, p < .01 (19 - 35 years);<br>6.5%, p < .01 (36-55 years);<br>5.5%, p < .01 (55-64 years);<br>**Uninsured:**<br>-4.9%, p < .01 (19 - 35 years);<br>-4.8%, p < .01 (36 - 55 years);<br>-4.0%, p < .01 (55 - 64 years);		**NS (2015);**<br>**NS (2016);**<br>**(Ref = 2013);**<br>P for interaction (year*age) NS<br><br>**Able to obtain needed prescriptions:**<br>**(Adjusted Prevalence Ratios)**<br>**Age 18-34 years (n=2,895)**<br>NS (2014);<br>NS (2015);<br>NS (2016);<br>(Ref = 2013);<br>**Age 35-44 years (n=1,672)**<br>NS (2014);<br>NS (2015);<br>NS (2016);<br>(Ref = 2013);<br>P for interaction (year*age) NS					

Andersen's domains		Measures of potential access			Measures of realized access				
**Type**	**Determinants/ factors**	**Insurance coverage/type**	**Usual source of care/regular primary care provider**	**Barriers to care, delayed, foregone care, unmet need**	**Utilization (use/non-use or frequency of use)**	**Preventive health visits (annual physical exam/routine checkup in last year)**	**Preventive health screening (blood pressure)**	**Preventive health screening (cholesterol screening)**	**Preventive health screening (Influenza shot)**
		**Individually Purchased Coverage**: NS (19 - 35 years); -1.3%, $p < .05$ (36 - 55 years); -1.8%, $p < .05$ (55 - 64 years). **Employer-Sponsored Coverage**: -1.4%, $p < .05$ (36 - 55 years); NS (36 - 55 years); NS (55 - 64 years); **Any Private Coverage**: -2.0%, $p < .01$ (19 - 35 years); NS (36 - 55 years); NS (55 - 64 years).							
	Children/has dependents	**Johnston (2018),** <u>**Post Medicaid Expansion**</u> **Uninsured**: -10.1%, $p < .05$ (Women with Dependent Children < 100% FPL); -27.4%, $p < .001$ (Women without Dependent Children < 100% FPL).	**Ahad (2019),** **Regular healthcare provider:** OR = 1.47, 95% CI [1.08-2.01], $p < .05$ (Children < 18 years). **Early (2018)** **Usual source of healthcare**	**Early (2018)** **Able to obtain timely medical care:** **(Adjusted Prevalence Ratios)** aPR = 0.91 [95% CI [0.85, 0.98] (Without children); (Ref = With children);	**Johnston (2018),** <u>**Post-Medicaid Expansion**</u> **Primary care visits in the last year:** NS (Women with Dependent Children < 100% FPL);	**DiPietro Mager (2021),** **Routine check-up past year:** NS (One or more children) (Ref = no children); **Margerison (2020),** <u>**Post Medicaid Expansion**</u>		**Margerison (2020),** <u>**Post Medicaid Expansion**</u> **Cholesterol check-in last year:** NS (Dependent children); NS (No dependent children).	

Andersen's domains		Measures of potential access			Measures of realized access				
**Type**	**Determinants/ factors**	**Insurance coverage/type**	**Usual source of care/regular primary care provider**	**Barriers to care, delayed, foregone care, unmet need**	**Utilization (use/non-use or frequency of use)**	**Preventive health visits (annual physical exam/routine checkup in last year)**	**Preventive health screening (blood pressure)**	**Preventive health screening (cholesterol screening)**	**Preventive health screening (Influenza shot)**
		**Margerison (2020),** **<u>Post-Medicaid Expansion</u>** **Has health coverage:** 7.6%, 95% CI [0.8, 14.4], p <.05 (Dependent children); 13.9%, 95% CI [8.7, 19.2], p <.05, (No dependent children).	**(Adjusted Prevalence Ratios)** aPR = 0.91 [95% CI [0.85, 0.98] (Without children); (Ref = With children). **Johnston (2018),** **<u>Post-Medicaid Expansion</u>** **No personal doctor**: NS (Women with Dependent Children < 100% FPL); -13.3%, p < .01 (Women without Dependent Children < 100% FPL).	**Able to obtain needed prescriptions:** **(Adjusted Prevalence Ratios)** aPR = 0.92 [95% CI [0.86, 0.98] (Without children); (Ref = With children). **Johnston (2018),** **<u>Post-Medicaid Expansion</u>** **Needed but could not see a doctor due to cost:** NS (Women with Dependent Children < 100% FPL); NS (Women without Dependent Children < 100% FPL); **Margerison (2020),** **<u>Post-Medicaid Expansion</u>** **Avoided seeking care due to cost:** –6.7%, 95% CI	NS (Women without Dependent Children < 100% FPL).	**Check-up in last year:** 4.6%, 95% CI [1.3, 7.9], p <.05 (Dependent children); 7.2%, 95% CI [2.5, 11.9], p <.05 (No dependent children).			

Andersen's domains		Measures of potential access			Measures of realized access				
Type	Determinants/ factors	Insurance coverage/type	Usual source of care/regular primary care provider	Barriers to care, delayed, foregone care, unmet need	Utilization (use/non-use or frequency of use)	Preventive health visits (annual physical exam/routine checkup in last year)	Preventive health screening (blood pressure)	Preventive health screening (cholesterol screening)	Preventive health screening (Influenza shot)
				[−11.9, −1.5], p <.05 (Dependent children); -10.5%, 95% CI [−16.2, −4.8], p <.05 (No dependent children).					
	Education	**Chen (2020)** <u>**Gained Medicaid Coverage in 2014 in Expansion States (N=149)**</u> **Uninsured in 2013:** NS (High school degree); NS (Some college); NS (College degree) (Ref = Less than High School). **Jones & Sonfield (2016),** <u>**Post Medicaid Expansion**</u> **Uninsured:** NS (High School); OR = 0.49, 95% CI [0.34–0.71], p < .001, (Some college); OR = 0.22, 95% CI [0.14–0.33], p	**Ahad (2019),** **Regular healthcare provider**: OR = 3.92, 95% CI [1.35-2.70], p < .05 (> High school education). **Early (2018)** **Usual source of healthcare** **(Adjusted Prevalence Ratios)** NS (High School graduate); aPR = 1.12 [95% CI [1.02, 1.22](Some college); NS (College graduate or more); (Ref = Less than high school).	**Early (2018)** **Able to obtain timely medical care:** **(Adjusted Prevalence Ratios)** aPR = 0.90 [95% CI [0.85, 0.96] (High School graduate); aPR = 0.86 [95% CI [0.80, 0.92] (Some college); aPR = 0.86 [95% CI [0.78, 0.94] (College graduate or more); (Ref = Less than high school); **Able to obtain needed prescriptions:** **(Adjusted Prevalence Ratios)**	**Farietta (2018),** <u>**Post Medicaid Expansion**</u> **Physician in the past year:** NS (High School); NS (Some college or associates degree); NS (Bachelor's degree or higher) (Ref = Less than high school). **Lee (2020),** **Family physician visits (past year):** -2.3%, 95% CI [-4.2, -0.4], p = .020 (High School); NS (Bachelors/graduate degree)	**DiPietro Mager (2021),** **Routine check-up past year:** NS (College graduate or >) (Ref = less than a college degree).			

Andersen's domains		Measures of potential access			Measures of realized access				
**Type**	**Determinants/ factors**	**Insurance coverage/type**	**Usual source of care/regular primary care provider**	**Barriers to care, delayed, foregone care, unmet need**	**Utilization (use/non-use or frequency of use)**	**Preventive health visits (annual physical exam/routine checkup in last year)**	**Preventive health screening (blood pressure)**	**Preventive health screening (cholesterol screening)**	**Preventive health screening (Influenza shot)**
		< .001 (Bachelors or higher)<br>(Ref - Less than high school).	**Farietta (2018), <u>Post Medicaid Expansion</u>**<br>**Usual source of care**: NS (High School);<br>NS (Some college or associates degree);<br>NS (Bachelor's degree or higher)<br>(Ref = Less than high school).	NS (High School graduate);<br>NS (Some college);<br>NS (College graduate or more);<br>(Ref = Less than high school).<br>**Farietta (2018)**<br>**<u>Post Medicaid Expansion</u>**<br>**Unmet healthcare need, mental health**:<br>OR = 0.51, 95% CI [0.29, 0.93], p < .05 (High school);<br>NS (Some college, associate degree); OR = 0.35, 95% CI [0.17, 0.72], p < .01 (Bachelors or higher)<br>(Ref = Less than high school);<br>**Unmet healthcare need, prescription drugs**:<br>NS (High School);<br>NS (Some college or associates degree);	(Ref = Less than High School);<br>**NP/PA visits (past year)**:<br>NS (High School);<br>4.3%, 95% CI [2.7, 5.9], p < .001 (Bachelors/graduate)<br>(Ref = Less than High School).<br>**Seo (2019),**<br>**Doctors visits in the past year:**<br>OR = 2.351, 95% CI [1.25, 4.42], p <0.001 (All women, High school graduate or GED);<br>OR = 2.916, 95% CI [1.13, 7.50], p = .030 (NBWA, High school graduate or GED);<br>OR = 2.589, 95% CI [1.02, 6.57], p = .050 (FBAI, High school graduate or GED);				

Andersen's domains		Measures of potential access			Measures of realized access				
Type	Determinants/ factors	Insurance coverage/type	Usual source of care/regular primary care provider	Barriers to care, delayed, foregone care, unmet need	Utilization (use/non-use or frequency of use)	Preventive health visits (annual physical exam/routine checkup in last year)	Preventive health screening (blood pressure)	Preventive health screening (cholesterol screening)	Preventive health screening (Influenza shot)
				NS (Bachelor's degree or higher) (Ref = Less than high school). **SteelFisher (2019), Avoided doctor due to discrimination concerns:** OR = 2.38, p < .05, 95% CI [1.00, 5.63] (College+) (Ref < College).	NS (All women, Bachelor and above); NS (NBWA, Bachelor and above); NS (FBAI, Bachelor and above) (Ref = less than high school).				
	**Employment status**	**Jones & Sonfield (2016), <u>Post Medicaid Expansion</u> Uninsured**: NS (1-34 hours); OR = 0.57, 95% CI [0.43–0.76], p < .001 (35 hours or more) (Ref - Not employed).	**Ahad (2019), Regular healthcare provider:** NS (Employed). **Farietta (2018), <u>Post Medicaid Expansion</u> Usual source of care:** NS (Working) (Ref = Not working).	**Farietta (2018), <u>Post Medicaid Expansion</u> Unmet healthcare need, mental health:** OR = 0.65, 95% CI [0.44, 0.97], p < .05 (Working) (Ref = Not working). **Unmet healthcare need, prescription drugs**: OR = 0.73, 95% CI [0.54, 0.98], p < .05 (Working) (Ref = Not working).	**Farietta (2018), <u>Post Medicaid Expansion</u> Physician in the past year:** NS (Working) (Ref = Not working). **Lee (2020), Family physician visits (past year):** NS (Employed); **NP/PA visits (past year):** NS (Employed). **Seo (2019), <u>Doctors visits in the past year</u>:**	**DiPietro Mager (2021), Routine check-up past year**: NS (Not currently employed) (Ref = Currently employed).			

Andersen's domains		Measures of potential access			Measures of realized access				
**Type**	**Determinants/ factors**	**Insurance coverage/type**	**Usual source of care/regular primary care provider**	**Barriers to care, delayed, foregone care, unmet need**	**Utilization (use/non-use or frequency of use)**	**Preventive health visits (annual physical exam/routine checkup in last year)**	**Preventive health screening (blood pressure)**	**Preventive health screening (cholesterol screening)**	**Preventive health screening (Influenza shot)**
					NS (All women, Unemployed); NS (NBWA, Unemployed); NS (FBAI, Unemployed); NS (All women, Self-employed or family business); NS (NBWA, Self-employed or family business); NS (FBAI, Self-employed or family business) (Ref = Employed by others).				
	**English proficiency**				**Seo (2019), <u>Doctors' visits in the past year</u>:** NS (All women, Well or very well); N/A (NBWA); NS (FBAI, Well or very well) (Ref = not well or not at all).				

Andersen's domains		Measures of potential access			Measures of realized access				
**Type**	**Determinants/ factors**	**Insurance coverage/type**	**Usual source of care/regular primary care provider**	**Barriers to care, delayed, foregone care, unmet need**	**Utilization (use/non-use or frequency of use)**	**Preventive health visits (annual physical exam/routine checkup in last year)**	**Preventive health screening (blood pressure)**	**Preventive health screening (cholesterol screening)**	**Preventive health screening (Influenza shot)**
	**Gender** identity and/or sexual orientation	**Dai (2019),** **No healthcare coverage:** **Sexual orientation** NS (Lesbian); NS (Bisexual); NS (Nonresponse group) AOR = 2.5, 95% CI [1.3, 4.6], p < .05 (Other) (Ref = straight women).	**Dai (2019),** **No personal care provider:** **Sexual orientation** NS (Lesbian); NS (Bisexual); NS (Nonresponse group); AOR = 2.4, 95% CI [1.3, 4.4], p < .05 (Other) (Ref = straight women).	**Dai (2019), Could not afford a doctor because of cost (past year):** **Sexual orientation** NS (Lesbian); NS (Bisexual);NS (Nonresponse group); NS (Other) (Ref = straight women). **SteelFisher (2019), Avoided doctor due to discrimination concerns:** **Sexual orientation/Gender identity** NS (LGBTQ) (Ref = non-LGBTQ).		**Dai (2019), Routine checkup in the past year:** **Sexual orientation** NS (Lesbian); NS (Bisexual); NS (Nonresponse group); NS (Other) (Ref = straight women).			**Dai (2019),** **Flu shot in the past year:** **Sexual orientation** AOR = 1.3, 95% CI [1.0, 1.6] , p < .05 (Lesbian); NS (Bisexual); NS (Nonresponse group); NS (Other) (Ref = straight women).
	**Health Literacy**		**Ahad (2019), Regular healthcare provider:** OR = 1.11, 95% CI [1.02-1.30], p < .05 (Health Literacy Scale, range 4-20).						
	**Citizenship status**		**Ahad (2019), Regular**	**Early (2018)** **Post-Medicaid Expansion**	**Seo (2019), Doctors visit in past year**:				

**Andersen's domains**		**Measures of potential access**			**Measures of realized access**				
**Type**	**Determinants/ factors**	**Insurance coverage/type**	**Usual source of care/regular primary care provider**	**Barriers to care, delayed, foregone care, unmet need**	**Utilization (use/non-use or frequency of use)**	**Preventive health visits (annual physical exam/routine checkup in last year)**	**Preventive health screening (blood pressure)**	**Preventive health screening (cholesterol screening)**	**Preventive health screening (Influenza shot)**
	**(immigrant status)**		**healthcare provider:**<br>OR = .27 (OR), 95% CI [.08-.91], p < .05 (African immigrant).<br><br>**Early (2018)**<br>**Post-Medicaid Expansion**<br>**Has Usual Source of Care:**<br>**(Adjusted Prevalence Ratios)**<br>**US-born or naturalized citizen (n=3,164)**<br>NS (2014);<br>NS (2015);<br>NS (2016);<br>(Ref = 2013);<br><br>Non-citizen (n=1,403)<br>NS (2014);<br>NS (2015);<br>NS (2016);<br>(Ref = 2013).<br>P for interaction (year*citizenship status) – NS.	**Able to obtain timely medical care:**<br>**(Adjusted Prevalence Ratios)**<br>**US-born or naturalized citizen (n=3,164)**<br>NS (2014);<br>NS (2015);<br>NS (2016);<br>(Ref = 2013);<br><br>**Non-citizen (n=1,403)**<br>NS (2014);<br>NS (2015);<br>NS (2016);<br>(Ref = 2013);<br>P for interaction (year*citizenship status) – NS;<br><br>**Able to obtain needed prescriptions:**<br>**(Prevalence Ratios)**<br>**US-born or naturalized citizen (n=3,164)**<br>NS (2014);	88.6% (NBWA); 77.9% (FBAI) (At least one visit);<br>11.4% (NBWA); 22.1% (FBAI) (No visit);<br>(comparison between groups, p <0.001).				

Andersen's domains		Measures of potential access			Measures of realized access				
Type	Determinants/ factors	Insurance coverage/type	Usual source of care/regular primary care provider	Barriers to care, delayed, foregone care, unmet need	Utilization (use/non-use or frequency of use)	Preventive health visits (annual physical exam/routine checkup in last year)	Preventive health screening (blood pressure)	Preventive health screening (cholesterol screening)	Preventive health screening (Influenza shot)
				NS (2015);<br>NS (2016);<br>(Ref = 2013).<br>**Non-citizen (n=1,403)**<br>NS (2014);<br>NS (2015);<br>NS (2016);<br>(Ref = 2013).<br>P for interaction (year*citizenship status) – NS.					
	**Information sources**		**Ahad (2019), Regular healthcare provider:**<br>OR = .32, 95% CI [.11-.89], p < .05 (respondent is the individual in the household most likely to obtain information to make health decisions).						
	**Marital status**	**Chen (2020),**<br>**<u>Gained Medicaid Coverage in 2014 in Expansion States (N=149)</u>**<br>**Uninsured in 2013:**<br>NS (Married)	**Ahad (2019), Regular healthcare provider**:<br>NS (Married).	**Margerison (2020),**<br>**<u>Post-Medicaid Expansion</u>**<br>**Avoided seeking care due to cost:**<br>–8.5%, 95% CI [–14.4, –2.6], p <.05 (Married);	**Lee (2020), Family physician visits (past year):**<br>1.5%, 95% CI [0.1, 2.9], p = .035 (Married);	**DiPietro Mager (2021), Routine check-up past year:**<br>NS (Married);<br>(Ref = not married).<br>**Margerison (2020),**		**Margerison (2020),**<br>**<u>Post Medicaid Expansion</u>**<br>**Cholesterol check-in last year:**<br>NS (Married);	

Andersen's domains		Measures of potential access			Measures of realized access				
Type	Determinants/ factors	Insurance coverage/type	Usual source of care/regular primary care provider	Barriers to care, delayed, foregone care, unmet need	Utilization (use/non-use or frequency of use)	Preventive health visits (annual physical exam/routine checkup in last year)	Preventive health screening (blood pressure)	Preventive health screening (cholesterol screening)	Preventive health screening (Influenza shot)
		(Ref = Not married).<br>**Jones & Sonfield (2016),**<br>**<u>Post Medicaid Expansion</u>**<br>**Uninsured**:<br>OR = 0.71, 95% CI [0.55–0.92], p = .009 (married)<br>(Ref - unmarried).<br>**Margerison (2020),**<br>**<u>Post-Medicaid Expansion</u>**<br>**Has healthcare coverage:**<br>11.9%, 95% CI [4.1, 19.7], p <.05 (Married);<br>7.6%, 95% CI [2.2, 12.9], p <.05<br>(Not married).		−6.8%, 95% CI<br>[−11.5, −2.2], p <.05 (Not married).	**NP/PA visits in past year:**<br>NS (Married).<br>**Seo (2019),**<br>**Doctor visit in the past year:**<br>NS (All women, Married or living together); NS (NBWA, Married or living together); NS (FBAI, Married or living together) (Ref = Widowed/separated/divorced or never married).	**<u>Post Medicaid Expansion</u>**<br>**Check-up last year:**<br>6.2%, 95% CI [1.3, 11.2], p <.05(Married); 4.5%, 95% CI [1.3, 7.9], p <.05 (Not married).		NS (Not married).	
	Number of births/Family size	**Jones & Sonfield (2016),**<br>**<u>Post Medicaid Expansion</u>**<br>**Uninsured:**<br>OR = 0.66, 95% CI [0.50, 0.89], p = .006 (1 birth); OR = 0.63, 95% CI [0.47–0.86], p = .003 (2 births)			**Lee (2020),**<br>**Family physician visits (past year):**<br>-0.7%, 95% CI<br>[-1.1, -0.3], p < .001 (Family size);<br>**NP/PA visits (past year):**<br>-1.4%, 95% CI				

Andersen's domains		Measures of potential access			Measures of realized access				
Type	Determinants/ factors	Insurance coverage/type	Usual source of care/regular primary care provider	Barriers to care, delayed, foregone care, unmet need	Utilization (use/non-use or frequency of use)	Preventive health visits (annual physical exam/routine checkup in last year)	Preventive health screening (blood pressure)	Preventive health screening (cholesterol screening)	Preventive health screening (Influenza shot)
		(Ref = 0 births).			[-1.8, -1.1], p < .001 (Family size).				
	Race and/or ethnicity	**Chen (2020),** <u>**Gained Medicaid Coverage in 2014 in Expansion States (N=149)**</u> **Uninsured in 2013:** OR = 0.14, 95% CI [0.02, 0.97], p = 0.046 (Black); OR=0.12, 95% CI [0.03, 0.49], p = 0.003 (Hispanic); NS (Other); [Ref = White]. **Jones & Sonfield (2016),** <u>**Post Medicaid Expansion**</u> **Uninsured:** NS (Black, Non-Hispanic); NS (Other, Non-Hispanic); OR = 2.2, 95% CI [1.67, 2.9], p < .001 (Hispanic, born in U.S.); OR = 4.57, 95% CI [3.37, 6.20], p < .001 (Hispanic,	**Early (2018)** **Usual source of healthcare** **(Adjusted Prevalence Ratios)** NS (Latina); NS (Non-Latina Black); NS (Non-Latina Asian/PI); aPR = 1.18 [95% CI [1.06, 1.32] (Non-Latina other/Multiple) (Ref = Non-Latina White). **Farietta (2018),** <u>**Post Medicaid Expansion**</u> **Usual source of care**: NS (Non-Hispanic black); NS (Other) (Ref = Non-Hispanic White).	**Early (2018)** **Able to obtain timely medical care:** **(Adjusted Prevalence Ratios)** NS (Latina); aPR = 1.11 [95% CI [1.02, 1.21] (Non-Latina Black); NS (Non-Latina Asian/PI); NS (Non-Latina other/Multiple) (Ref = Non-Latina White); **Able to obtain needed prescriptions:** **(Adjusted Prevalence Ratios)** NS (Latina); NS (Non-Latina Black); NS (Non-Latina Asian/PI); NS (Non-Latina other/Multiple)	**Farietta (2018),** <u>**Post Medicaid Expansion**</u> **Physician visit in the past year**: OR = 1.54, 95% CI [1.06, 2.25], p < .05 (Non-Hispanic black); NS (Other) (Ref = Non-Hispanic White). **Lee (2020),** **Family physician visits (past year)**: -2.5%, 95% CI [-4.1, -0.9], p = .002, (Black, Non-Hispanic); -3.1%, 95% CI [-4.8, -1.4], p < .001 (Other race, Non-Hispanic); -3.4%, 95% CI [-5.2,-1.5], p < .001(Hispanic)		**Pazol (2017),** **BP checked by healthcare professional in the past year:** 84.9%, 95% CI [83.3–86.4] (Non-Hispanic white); 82.5%, 95% CI [79.6–85.2] (Non-Hispanic black); 69.4%, 95% CI [66.5–72.1] (Hispanic); 78.8%, 95% CI [74.9–82.3] (Non-Hispanic other or multiple races).		**Pazol (2017),** <u>**Received an influenza vaccine in the past year**</u>**:** 32.8%, 95% CI [30.9–34.7] (Non-Hispanic white); 28.0%, 95% CI [24.8–31.3] (Non-Hispanic black); 27.6%, 95% CI [25.1–30.2] (Hispanic); 39.9%, 95% CI [35.7–44.4] (Non-Hispanic other or multiple races).

Andersen's domains		Measures of potential access			Measures of realized access				
Type	Determinants/ factors	Insurance coverage/type	Usual source of care/regular primary care provider	Barriers to care, delayed, foregone care, unmet need	Utilization (use/non-use or frequency of use)	Preventive health visits (annual physical exam/routine checkup in last year)	Preventive health screening (blood pressure)	Preventive health screening (cholesterol screening)	Preventive health screening (Influenza shot)
		not born in the U.S.)<br>(Ref = White, Non-Hispanic).<br>**Lee (2018),**<br>**<u>Post Medicaid Expansion,</u>**<br>**<u>(Difference in percentage-point change between FPL groups),</u>**<br>**Uninsured:**<br>**≤ 138% FPL**<br>**Race**<br>−14.67%, 95% CI [−16.43, −12.91] (White);<br>−13.89%, 95% CI [−16.35, −11.44] (Black);<br>−14.21%, 95% CI [−18.67, −9.76] (Other);<br>**Ethnicity**<br>−15.2%, 95% CI [−16.79, −13.61] (Hispanic);<br>−13.47%, 95% CI [−16.07, −10.86] (Non-Hispanic);<br>**139–399% FPL:**<br>**Race:**		(Ref = Non-Latina White).<br><br>**Farietta (2018),**<br>**<u>Post Medicaid Expansion</u>**<br>**Unmet healthcare needs, mental health**:<br>NS (Non-Hispanic black);<br>NS (Other)<br>(Ref = Non-Hispanic White).<br>**Unmet healthcare needs, prescription drugs**:<br>NS (Non-Hispanic black);<br>NS (Other)<br>(Ref = Non-Hispanic White).<br>**SteelFisher (2019),**<br>**Avoided doctor due to discrimination concerns:**<br>OR = 3.69, 95% CI [1.59, 8.58], $p < .05$ (Hispanic/Latina);<br>OR 5.97, 95% CI [2.00, 17.87], p <	(Ref = White, Non-Hispanic);<br>**NP/PA visits (past year)**:<br>-6.7%, 95% CI [-8.2, -5.1], $p < .001$ (Black, Non-Hispanic);<br>-6.4%, 95% CI<br>[-7.8, -5], $p < .001$ (Other Race, Non-Hispanic);<br>-6.7%, 95% CI<br>[-8.7, -4.7], $p < .001$(Hispanic)<br>(Ref = White, Non-Hispanic).<br>**Seo (2019), Doctors visits in the past year:**<br>OR = 0.537, 95% CI [0.35–0.84], p = .008 (FBAI)<br>(Ref = NBWA).				

Andersen's domains		Measures of potential access			Measures of realized access				
Type	Determinants/ factors	Insurance coverage/type	Usual source of care/regular primary care provider	Barriers to care, delayed, foregone care, unmet need	Utilization (use/non-use or frequency of use)	Preventive health visits (annual physical exam/routine checkup in last year)	Preventive health screening (blood pressure)	Preventive health screening (cholesterol screening)	Preventive health screening (Influenza shot)
		−8.20%, 95% CI [−9.29, −7.12] (White); −8.35%, 95% CI [−10.17, −6.52] (Black); −14.16%, 95% CI [−16.64, −11.68] (Other); **Ethnicity:** −8.11%, 95% CI [−9.07, −7.15] (Hispanic); −14.19%, 95% CI [−16.42, −11.96] (Non-Hispanic); **<u>Difference in percentage-point change between FPL groups,</u>** **≤ 138% FPL vs. 139–399% FPL:** **Race:** −6.47%, 95% CI [−8.57, −4.36] (White); −5.55%, 95% CI [-8.56, −2.53] (Black); NS (Other); **Ethnicity:** −7.09%, 95% CI [−9.02, −5.17] (Hispanic);		0.5 (Native American); NS (Black); NS (Asian) (Ref =White).					

Andersen's domains		Measures of potential access			Measures of realized access				
Type	Determinants/ factors	Insurance coverage/type	Usual source of care/regular primary care provider	Barriers to care, delayed, foregone care, unmet need	Utilization (use/non-use or frequency of use)	Preventive health visits (annual physical exam/routine checkup in last year)	Preventive health screening (blood pressure)	Preventive health screening (cholesterol screening)	Preventive health screening (Influenza shot)
		NS (Non-Hispanic).<br>**Wehby (2018),**<br>**Post Medicaid Expansion**<br>**Medicaid Insurance**:<br>5.7%, $p < .01$ (White);<br>6.6%, $p < .01$ (Black);<br>6.0%, $p < .01$ (Other);<br>7.5%, $p < .01$ (Hispanic);<br>**Uninsured**:<br>-3.9%, $p < .01$ (White);<br>-3.8%, $p < .01$ (Black);<br>-4.1%, $p < .05$ (Other);<br>-5.1%, $p < .01$ (Hispanic);<br>**Individually Purchased Coverage**:<br>-0.8%, $p < .05$ (White);<br>NS (Black);<br>-3.1%, $p < .05$ (Other);<br>NS (Hispanic);							

Andersen's domains		Measures of potential access			Measures of realized access				
Type	Determinants/ factors	Insurance coverage/type	Usual source of care/regular primary care provider	Barriers to care, delayed, foregone care, unmet need	Utilization (use/non-use or frequency of use)	Preventive health visits (annual physical exam/routine checkup in last year)	Preventive health screening (blood pressure)	Preventive health screening (cholesterol screening)	Preventive health screening (Influenza shot)
		**Employer-Sponsored Coverage**:<br>NS (White);<br>NS (Black);NS (Other);<br>NS (Hispanic);<br>**Any Private Coverage:**<br>-1.7%, p < .01 (White);<br>-2.5%, p < .01 (Black);<br>NS (Other);<br>-2.4%, p < .05 (Hispanic).							
**Individual**<br>**Enabling factors**	**Income**	**Jones & Sonfield (2016),**<br>**<u>Post Medicaid Expansion</u>**<br>**Uninsured:**<br>OR = 2.12, 95% CI [1.67, 2.69], p < .001 (≤138% FPL)<br>(Ref = 139% or >).<br>**<u>Lee (2018),</u>**<br>**<u>Post Medicaid Expansion,</u>**<br>**<u>(Percent difference before versus after ACA (percent change),</u>**<br>**Insurance status:**	**Early (2018)**<br>**Usual source of healthcare**<br>**(Adjusted Prevalence Ratios)**<br>NS (51-100% of poverty);<br>NS (101-138% of poverty);<br>(Ref = 0-50% of poverty).	**Early (2018)**<br>**Able to obtain timely medical care:**<br>**(Adjusted Prevalence Ratios)**<br>NS (51-100% of poverty);<br>NS (101-138% of poverty);<br>(Ref = 0-50% of poverty);<br><br>**Able to obtain needed prescriptions:**	**<u>Lee (2019),</u>**<br>**<u>Post-Medicaid Expansion</u>**<br>**<u>(Percent difference after ACA, percent change),</u>**<br>**Seen/talked to a doctor in 12 months:**<br>4.10%, 95% CI [2.70, 5.49], p < .05 (≤138% FPL);<br>2.31%, 95% CI [1.12, 3.51], p < .05 (139%–399% FPL);		**Pazol (2017),**<br>**BP checked by healthcare professional in the past year:**<br>72.9%, 95% CI [70.5–75.3] (≤138% FPL);<br>77.3%, 95% CI [74.3–80.1] (139%–250% FPL);<br>83.0, 95% CI [80.1–85.5] (251%–400% FPL);<br>89.4%, 95% CI [87.4–91.2] (>400% FPL).	**Lee (2019),**<br>**<u>Post-Medicaid Expansion</u>**<br>**<u>(Percent difference after ACA, percent change),</u>**<br>**Had Cholesterol screening (45-64 years):**<br>7.11%, 95% CI [4.86, 9.35], p < .05 (≤138% FPL);<br>4.71%, 95% CI [3.11, 6.31], p < .05 (139%–399% FPL);	**Pazol (2017), Influenza vaccine in the past year:**<br>24.2%, 95% CI [22.2–26.4] (≤138% FPL);<br>28.6%, 95% CI [25.8–31.6] (139%–250% FPL);<br>31.0%, 95% CI [28.2–34.0] (251%–400% FPL);<br>41.8%, 95% CI [38.9–44.7] (>400% FPL).<br>**Lee (2019),**

Andersen's domains		Measures of potential access			Measures of realized access				
**Type**	**Determinants/ factors**	**Insurance coverage/type**	**Usual source of care/regular primary care provider**	**Barriers to care, delayed, foregone care, unmet need**	**Utilization (use/non-use or frequency of use)**	**Preventive health visits (annual physical exam/routine checkup in last year)**	**Preventive health screening (blood pressure)**	**Preventive health screening (cholesterol screening)**	**Preventive health screening (Influenza shot)**
		**Private:**<br>4.36%, 95% CI [2.77, 5.95] (≤138% FPL);<br>3.60%, 95% CI [2.38, 4.83] (139–399% FPL);<br>NS (≥ 400% FPL);<br>**Medicare/Medicaid/Other Public:**<br>9.61%, 95% CI [7.75, 11.47] (≤138% FPL);<br>4.73%, 95% CI [3.83, 5.63] (139–399% FPL);<br>0.35%, 95% CI [−0.19, 0.89] (≥ 400% FPL);<br>**Uninsured:**<br>-14.5%, 95% CI<br>[-15.9, -13.1] (≤138% FPL);<br>−8.74%, 95% CI [−9.63, − 7.85] (139–399% FPL);<br>1.92%, 95% CI [−2.34, −1.50] (≥ 400% FPL);<br>**Difference in percentage point change between FPL groups**<br>**Insurance status:**		**(Adjusted Prevalence Ratios)**<br>NS (51-100% of poverty);<br>NS (101-138% of poverty);<br>(Ref = 0-50% of poverty).<br>**Lee (2018),**<br>**Post-Medicaid Expansion (Percent difference before versus after ACA (percent change),**<br>**Affordability:**<br>**Delayed care:**<br>-5.93%, 95% CI<br>[-7.16, -4.71]<br>(≤138% FPL);<br>−3.44%, 95% CI [−4.28, −2.60] (139–399% FPL);<br>-1.57%, 95% CI [−2.09, −1.04<br>(≥ 400% FPL);<br>**Did not get care:**<br>-5.04%, 95% CI<br>[-6.25, -3.83] (≤138% FPL);<br>−3.05%, 95% CI [−3.76, −2.34] (139–399% FPL);	NS, (≥400% FPL).<br>**Lee (2020),**<br>**Family physician visits (past year):**<br>NS (Middle income);<br>NS (High income)<br>(Ref = low income).<br>**NP/PA visits (past year):**<br>NS (Middle income);<br>NS (High income)<br>(Ref = low income).<br>**Seo (2019), Doctors visits in the past year:**<br>OR = 0.654, 95% CI [0.45–0.94], p = .026 (All women, 139%–399%);<br>NS (NBWA, 139%–399%);<br>NS (FBAI, 139%–399% FPL);		**Lee (2019),**<br>**Post-Medicaid Expansion**<br>**(Percent difference after ACA, percent change),**<br>**Had BP screening:**<br>4.45%, 95% CI [3.21, 5.69], p < .05 (≤138% FPL);<br>2.92%, 95% CI [2.02, 3.81], p < .05 (139%–399%);<br>1.04%, 95% CI [0.32, 1.75], p < .05 (≤138% FPL vs ≥400% FPL).	1.78%, 95% CI [0.26, 3.30], p < .05 (≤138% FPL vs ≥400% FPL).	**Post Medicaid Expansion**<br>**(Percent difference after ACA, percent change),**<br>**Receipt of Influenza immunization**:<br>5.55%, 95% CI [4.17, 6.94], p < .05 (≤138% FPL);<br>4.17%, 95% CI [3.01, 5.34], p < .05, (139%–399% FPL);<br>3.47%, 95% CI [2.20, 4.73], p < .05 (≤138% FPL vs ≥400% FPL).

Andersen's domains		Measures of potential access			Measures of realized access				
Type	Determinants/ factors	Insurance coverage/type	Usual source of care/regular primary care provider	Barriers to care, delayed, foregone care, unmet need	Utilization (use/non-use or frequency of use)	Preventive health visits (annual physical exam/routine checkup in last year)	Preventive health screening (blood pressure)	Preventive health screening (cholesterol screening)	Preventive health screening (Influenza shot)
		**Private:** 2.84%, 95% CI [1.07, 4.60] (≤ 138% FPL vs. ≥ 400% FPL); **Medicare/Medicaid/Other Public**: 9.26%, 95% CI [7.36, 11.16] (≤ 138% FPL vs. ≥ 400% FPL); **Uninsured**: -12.59%, 95% CI [−14.12, −11.07] (≤ 138% FPL vs. ≥ 400% FPL).		−1.09%, 95% CI [−1.46, −0.71] (≥ 400% FPL); **Problems paying medical bills:** -5.64%, 95% CI [-7.42, -3.86] (≤138% FPL); −4.36%, 95% CI [−5.56, −3.17] (139–399% FPL); -1.07%, 95% CI [-1.82, −0.31] (≥ 400% FPL); **Difference in percentage point change between FPL groups,** **Affordability:** **Delayed care**: −4.37%, 95% CI [−5.73, −3.00] (≤ 138% FPL vs. ≥ 400% FPL); **Did not get care**: −3.95%, 95% CI [-5.24, −2.66] (≤ 138% FPL vs. ≥ 400% FPL); **Problems paying medical bills:** −4.57%, 95% CI	NS (All women, 400% FPL or >); NS (NBWA, 400% FPL or >); NS (FBAI or 400% FPL or >) (Ref = 0%–138% FPL).				

Andersen's domains		Measures of potential access			Measures of realized access				
**Type**	**Determinants/ factors**	**Insurance coverage/type**	**Usual source of care/regular primary care provider**	**Barriers to care, delayed, foregone care, unmet need**	**Utilization (use/non-use or frequency of use)**	**Preventive health visits (annual physical exam/routine checkup in last year)**	**Preventive health screening (blood pressure)**	**Preventive health screening (cholesterol screening)**	**Preventive health screening (Influenza shot)**
				[-6.49, −2.64] (≤ 138% FPL vs. ≥ 400% FPL).<br>**Lee (2019),**<br>**Post Medicaid Expansion**<br>**(Percent difference after ACA, percent change)**<br>**Insurance affordability (difficult to find affordable insurance):**<br>-25.24%, 95% CI [−32.79, −17.68], p < .05 (≤138% FPL);<br>−13.28%, 95% CI [−17.37, −9.19], p < .05 (139%–399% FPL);<br>NS (≥400% FPL).<br>**SteelFisher (2019),**<br>**Avoided doctor due to discrimination concerns:**<br>NS ($25k-<50k);<br>NS ($50k-<75k); NS ($75k+)<br>(Ref = $<25k).					

Andersen's domains		Measures of potential access			Measures of realized access				
Type	Determinants/ factors	Insurance coverage/type	Usual source of care/regular primary care provider	Barriers to care, delayed, foregone care, unmet need	Utilization (use/non-use or frequency of use)	Preventive health visits (annual physical exam/routine checkup in last year)	Preventive health screening (blood pressure)	Preventive health screening (cholesterol screening)	Preventive health screening (Influenza shot)
	**Insurance (no insurance, type of insurance)**		**Ahad (2019),**<br>**Regular healthcare provider:**<br>OR = .20, 95% CI [.06-.70], p < .05 (Medicaid or Medi-Cal health insurance);<br>NS (No health insurance).<br>**Early (2018)**<br>**<u>Post-Medicaid Expansion</u>**<br>**Has Usual Source of Care:**<br>**(Adjusted Prevalence Ratios)**<br>**<u>Type of Health Insurance Coverage</u>**<br>aPR = 1.43 [95% CI [1.26, 1.62] (Medi-Cal);<br>aPR = 1.55 [95% CI [1.37, 1.75] (Private);<br>aPR = 1.39 [95% CI [1.16, 1.66] (Other Public)<br>(Ref = Uninsured).<br><br>**Early (2018)**	**Early (2018)**<br>**<u>Post-Medicaid Expansion</u>**<br>**Able to obtain timely medical care:**<br>**(Adjusted Prevalence Ratios)**<br>**Type of health insurance coverage**<br>NS (Medi-Cal);<br>NS (Private);<br>NS (Public);<br>Ref = No insurance).<br>**Able to obtain needed prescriptions**<br>NS (Medi-Cal);<br>NS (Private);<br>NS (Public);<br>Ref = No insurance).<br><br>**Early (2018)**<br>**Post-Medicaid Expansion**<br>**Able to obtain timely medical care:**	**Lee (2020),**<br>**<u>Family physician visits (past year)</u>:**<br>11.7%, 95% CI [10.2, 13.2], p < .001 (Health insurance);<br>**NP/PA visits (past year):**<br>4.8%, 95% CI [3.2, 6.4], p < .001 (Health insurance).<br>**Seo (2019),**<br>**Doctors visits in the past year:**<br>OR = 0.291,95% CI [0.16–0.52], p <0.001 (All women, uninsured);<br>OR = 0.321, 95% CI [0.17–0.61], p <0.001 (NBWA, uninsured);<br>OR = 0.245, 95% CI [0.08–0.79], p = .022 (FBAI, uninsured);<br>NS (All women, Public insurance);	**DiPietro Mager (2021),**<br>**Routine check-up past year:**<br>NS (Any insurance coverage)<br>(Ref = no insurance).	**Pazol (2017),**<br>**BP checked by a healthcare professional in the past year:**<br>87.3%, 95% CI [86.0–88.5] (Had insurance coverage continuously during the past year);<br>81.0%, 95% CI [77.4–84.1] (Had insurance coverage with gaps during the past year);<br>51.6%, 95% CI [47.7–55.4] (Did not have any insurance coverage during the past year).		**Pazol (2017),**<br>**Influenza vaccine in the past year:**<br>37.0%, 95% CI [35.4–38.6] (Had insurance coverage continuously during the past year);<br>24.0%, 95% CI [20.8–27.6] (Had insurance coverage with gaps during the past year);<br>14.1%, 95% CI [11.8–16.7] (Did not have any insurance coverage during the past year).

Andersen's domains		Measures of potential access			Measures of realized access				
**Type**	**Determinants/ factors**	**Insurance coverage/type**	**Usual source of care/regular primary care provider**	**Barriers to care, delayed, foregone care, unmet need**	**Utilization (use/non-use or frequency of use)**	**Preventive health visits (annual physical exam/routine checkup in last year)**	**Preventive health screening (blood pressure)**	**Preventive health screening (cholesterol screening)**	**Preventive health screening (Influenza shot)**
			**Post-Medicaid Expansion**<br>**Has Usual Source of Care:**<br>**<u>Type of Health Insurance Coverage</u>**<br>**(Adjusted Prevalence Ratios)**<br>**Medi-Cal**<br>NS (2014);<br>NS (2015);<br>NS (2016);<br>(Ref = 2013);<br>**Private**<br>NS (2014);<br>NS (2015);<br>NS (2016);<br>(Ref = 2013);<br>**Uninsured**<br>NS (2014);<br>NS (2015);<br>NS (2016);<br>(Ref = 2013);	**(Adjusted Prevalence Ratios)**<br>**Medi-Cal**<br>NS (2014);<br>NS (2015);<br>NS (2016);<br>(Ref = 2013);<br>**Private**<br>NS (2014);<br>NS (2015);<br>NS (2016);<br>(Ref = 2013);<br>**Uninsured**<br>NS (2014);<br>NS (2015);<br>NS (2016);<br>(Ref = 2013);<br>**Able to obtain needed prescriptions:**<br>**<u>Type of Health Insurance Coverage</u>**<br><br>**(Adjusted Prevalence Ratios)**<br>**Medi-Cal**<br>NS (2014);<br>NS (2015);<br>NS (2016);	NS (NBWA, Public insurance)<br>NS (FBAI, Public insurance);<br>NS (All women, Privately purchased insurance);<br>NS (NBWA, Privately purchased insurance)<br>NS (FBAI, Privately purchased insurance)<br>(Ref = Employment-based insurance).				

Andersen's domains		Measures of potential access			Measures of realized access				
Type	Determinants/ factors	Insurance coverage/type	Usual source of care/regular primary care provider	Barriers to care, delayed, foregone care, unmet need	Utilization (use/non-use or frequency of use)	Preventive health visits (annual physical exam/routine checkup in last year)	Preventive health screening (blood pressure)	Preventive health screening (cholesterol screening)	Preventive health screening (Influenza shot)
				(Ref = 2013);<br>**Private**<br>NS (2014);<br>NS (2015);<br>NS (2016);<br>(Ref = 2013);<br>**Uninsured**<br>NS (2014);<br>NS (2015);<br>NS (2016);<br>(Ref = 2013);<br>**SteelFisher (2019),**<br>**<u>Avoided doctor due to discrimination concerns</u>:**<br>NS (Medicaid);<br>OR = 8.57, 95% CI [2.91, 25.24], p < .05 (Uninsured); (Ref = Non-Medicaid).					
	Residence (Urban/Rural)				**Lee (2020),**<br>**Any medical provider visits (past year):**<br>NS (Rural/urban difference);<br>**Visits with primary care**				

Andersen's domains		Measures of potential access			Measures of realized access				
Type	Determinants/ factors	Insurance coverage/type	Usual source of care/regular primary care provider	Barriers to care, delayed, foregone care, unmet need	Utilization (use/non-use or frequency of use)	Preventive health visits (annual physical exam/routine checkup in last year)	Preventive health screening (blood pressure)	Preventive health screening (cholesterol screening)	Preventive health screening (Influenza shot)
					**providers (past year):** 4.2%, 95% CI [1.1, 7.3], p = .008 (Rural/urban difference); **Family physician visits (past year):** NS (Rural/urban difference); **NP/PAs visits (past year):** 8.5%, 95% CI [4.9, 12.1], p < .001 (Rural/urban difference).				
	**Access to Good Public Transit**		**Ahad (2019) Regular healthcare provider:** NS. (Access to good public transit).						
	**Usual Source of Care**				**Seo (2019), Doctors visits in the past year:** OR = 3.890, 95% CI 2.46, 6.15], p < .001 (All women, usual source of care);				

Andersen's domains		Measures of potential access			Measures of realized access				
**Type**	**Determinants/ factors**	**Insurance coverage/type**	**Usual source of care/regular primary care provider**	**Barriers to care, delayed, foregone care, unmet need**	**Utilization (use/non-use or frequency of use)**	**Preventive health visits (annual physical exam/routine checkup in last year)**	**Preventive health screening (blood pressure)**	**Preventive health screening (cholesterol screening)**	**Preventive health screening (Influenza shot)**
					OR = 4.637, 95% CI [2.77, 7.77], p < .001 (NBWA, usual source of care); OR = 2.970, 95% CI [1.20, 7.32], p = .021 (FBAI, usual source of care) (Ref = no usual source of care).				
**Individual Need factors**	**BMI > 25.0**		**Ahad (2019), Regular healthcare provider:** NS (BMI >25.0).						
	**Depression (PHQ2 score > 2.0)**		**Ahad (2019), Regular healthcare provider:** NS (HQ-2 depression score ≥ 2.0).						
	**History of chronic disease**				**Seo (2019), Doctors' visits in the past year:** OR = 1.907, 95% CI [1.16, 3.13], p = .013 (All women, with chronic disease); OR = 1.725, 95% CI [1.02,				

Andersen's domains		Measures of potential access			Measures of realized access				
Type	Determinants/ factors	Insurance coverage/type	Usual source of care/regular primary care provider	Barriers to care, delayed, foregone care, unmet need	Utilization (use/non-use or frequency of use)	Preventive health visits (annual physical exam/routine checkup in last year)	Preventive health screening (blood pressure)	Preventive health screening (cholesterol screening)	Preventive health screening (Influenza shot)
					2.93], p = .047 (NBWA, with chronic disease); OR = 2.394, 5% CI [1.07, 5.38], p = .038 (FBAI, with chronic disease) (Ref = Without chronic disease).				
	**History of mental health illness**	**Massetti (2017), Has healthcare coverage:** 79.9%, 95% CI [78.7, 81.1] (Mental Health problems); 80.5%, 95% CI [79.7, 81.3] (No Mental Health problems) (difference between groups NS).				**Massetti (2017), Had a check-up in 2 past years:** 79.5%, 95% CI [78.3, 80.6] (Mental Health problems); 82.2% 95% CI [81.5, 83.0] (No Mental Health problems) (difference between groups p < .001).			**Massetti (2017), Influenza vaccination in past year:** 31.9%, 95% CI [30.6, 33.3] (Mental Health problems); 33.6% 95% CI [32.7, 34.5] (No Mental Health problems), (difference between groups NS).
	**Hypertension**		**Ahad (2019), Regular healthcare provider:** OR = 8.57, 95% CI [1.45-50.73], p < .05 (High blood pressure).						

Andersen's domains		Measures of potential access			Measures of realized access				
**Type**	**Determinants/ factors**	**Insurance coverage/type**	**Usual source of care/regular primary care provider**	**Barriers to care, delayed, foregone care, unmet need**	**Utilization (use/non-use or frequency of use)**	**Preventive health visits (annual physical exam/routine checkup in last year)**	**Preventive health screening (blood pressure)**	**Preventive health screening (cholesterol screening)**	**Preventive health screening (Influenza shot)**
	**Self-assessed health**	**Chen (2020)** **<u>Gained Medicaid Coverage in 2014 in Expansion States (N=149)</u>** **Uninsured in 2013:** OR = 4.50, 95% CI [1.23, 16.48], p = 0.024 (Health status - Fair or poor) (Ref = Health status excellent, very good, or good).	**Ahad (2019), Regular healthcare provider:** NS (Self-assessed health ≥ Good).		**Lee (2020),** **Family physician visits (past year):** 11.5%, 95% CI [8.4, 14.5], p < .001 (Poor or fair health status); **NP/PA visits (past year):** 3.4%, 95% CI [1, 5.9], p = 0.006 (Poor or fair health status). **Seo (2019), Doctors visit in the past year:** NS (All women, Good or better); NS (NBWA, Good or better); NS (FBAI, Good or better) (Ref = Fair or poor).				
	**Psychological distress**			**Johnson (2020),** **Medical care delayed due to cost, past 12 months:**					

Andersen's domains		Measures of potential access			Measures of realized access				
Type	Determinants/ factors	Insurance coverage/type	Usual source of care/regular primary care provider	Barriers to care, delayed, foregone care, unmet need	Utilization (use/non-use or frequency of use)	Preventive health visits (annual physical exam/routine checkup in last year)	Preventive health screening (blood pressure)	Preventive health screening (cholesterol screening)	Preventive health screening (Influenza shot)
				AOR = 2.0, 95% CI [1.5, 2.6], p < .001 (Moderate distress); AOR = 2.9, 95% CI [2.0, 4.4], p < .001 (Severe distress) (Ref = no distress); **Needed but couldn't afford care, past 12 months:** AOR = 1.9, 95% CI [1.3, 2.6], p < .001 (Moderate distress); AOR = 4.1, 95% CI [2.7, 6.4]; p < .001 (Severe distress) (Ref = no distress). **Needed but couldn't afford mental health care, past 12 months:** AOR = 6.1, 95% CI [3.3, 11.2], p < .001 (Moderate distress); AOR = 19.7, 95% CI [10.2, 37.8], p < .001 (Severe distress) (Ref = no distress); **Needed but couldn't afford**					

Andersen's domains		Measures of potential access			Measures of realized access				
Type	Determinants/ factors	Insurance coverage/type	Usual source of care/regular primary care provider	Barriers to care, delayed, foregone care, unmet need	Utilization (use/non-use or frequency of use)	Preventive health visits (annual physical exam/routine checkup in last year)	Preventive health screening (blood pressure)	Preventive health screening (cholesterol screening)	Preventive health screening (Influenza shot)
				**prescription medicines, past 12 months:** AOR = 2.2, 95% CI [1.6, 2.9], p < .001 (Moderate distress); AOR = 4.1, 95% CI [2.7, 6.3], p < .001 (Severe distress) (Ref = no distress).					
	**Perceived mental health status**				**Lee (2020), Family physician visits (past year):** 6.4%, 95% CI [3, 9.9], p < .001 (Poor or fair mental health status); **NP/PA visits (past year):** NS (Poor or fair mental health status).				
**Health Behaviors**	**Tobacco Use**		**Ahad (2019), Regular healthcare provider:** NS (Tobacco use).						
**Contextual Predisposing factors**	**N/A**								

Andersen's domains		Measures of potential access			Measures of realized access				
**Type**	**Determinants/ factors**	**Insurance coverage/type**	**Usual source of care/regular primary care provider**	**Barriers to care, delayed, foregone care, unmet need**	**Utilization (use/non-use or frequency of use)**	**Preventive health visits (annual physical exam/routine checkup in last year)**	**Preventive health screening (blood pressure)**	**Preventive health screening (cholesterol screening)**	**Preventive health screening (Influenza shot)**
**Contextual Enabling factors**	**County unemployment rate**				**Lee (2020),** **Family physician visits (past year):** 0.5%, 95% CI [0.2, 0.8], p < .001; **NP/PA visits (past year):** -0.5%, 95% CI [-0.8, -0.2], p < .001.				
	**Family medicine physicians per 100,000 population**				**Lee (2020),** **Family physician visits (past year):** 0.2%, 95% CI [0.1, 0.3], p < .001; **NP/PA visits (past year):** 0.1%, 95% CI [0, 0.2], p = .002.				
	**Nurse practitioners and physician assistants per 100,000 population**				**Lee (2020),** **Family physician visits (past year):** NS; **NP/PA visits (past year):** 0.1%, 95% CI [0.1, 0.1], p < .001.				

Andersen's domains		Measures of potential access			Measures of realized access				
**Type**	**Determinants/ factors**	**Insurance coverage/type**	**Usual source of care/regular primary care provider**	**Barriers to care, delayed, foregone care, unmet need**	**Utilization (use/non-use or frequency of use)**	**Preventive health visits (annual physical exam/routine checkup in last year)**	**Preventive health screening (blood pressure)**	**Preventive health screening (cholesterol screening)**	**Preventive health screening (Influenza shot)**
	**Pre/post-implementation of ACA Medicaid expansion (various outcome measures)**	**Chen (2020),**<br>**Probability of insurance type in 2014 based on 2013 insurance type - Expansion State**<br>**Uninsured 2014:**<br>50.0%, p <.01 (Uninsured); 2.9%, p < 0.01 (Medicaid);<br>NS (Group);<br>**Medicaid 2014:**<br>38.7%, p < 0.01 (Uninsured); 94.6%, p < 0.01 (Medicaid);<br>NS (Group);<br>**Group (2014):**<br>NS (Uninsured); NS (Medicaid);<br>NS (Group);<br>**Probability of insurance type in 2014 based on 2013 insurance type - Non-Expansion State**<br>**Uninsured 2014:**<br>NS (Uninsured);<br>NS (Medicaid);<br>NS (Group);<br>**Medicaid 2014:**	**Daw (2019),**<br>**Post-Medicaid Expansion Years 2 and 3 (Post policy)**<br>**No Usual Source of Care:**<br>-2.4%, 95% CI [–4.5, –0.3].<br>**Early (2018)**<br>**Post-Medicaid Expansion**<br>**Has Usual Source of Care:**<br>**(Adjusted Prevalence Ratios)**<br>NS (2014);<br>NS (2015);<br>NS (2016);<br>(Ref = 2013);<br>**Type of Health Insurance Coverage**<br>1.43 (Medi-Cal);<br>1.55 (Private); 1.39 (Other Public)<br>(Ref = No insurance).<br>**Farietta (2018),**<br>**Post-Medicaid Expansion**	**Daw (2019),**<br>**Post-Medicaid Expansion Years 2 and 3 (Post policy)**<br>**Delayed or did not receive medical care because of cost:**<br>-1.5%, 5% CI [-2.6, -0.5].<br>**Early (2018)**<br>**Post-Medicaid Expansion**<br>**Able to obtain timely medical care:**<br>**(Adjusted Prevalence Ratios)**<br>NS (2014);<br>NS (2015);<br>NS (2016);<br>(Ref = 2013);<br>**Able to obtain needed prescriptions:**<br>NS (2014);<br>NS (2015);<br>NS (2016);<br>(Ref = 2013);<br>**Farietta (2018),**<br>**Post-Medicaid Expansion**	**Farietta (2018),**<br>**Post-Medicaid Expansion**<br>**Physician in the past year:**<br>NS.<br>**Johnston (2018),**<br>**Post-Medicaid Expansion**<br>**Primary care visit in the past year:**<br>NS.	**Margerison (2020),**<br>**Post Medicaid Expansion**<br>**Check-up in last year:**<br>5.1%, 95% CI [1.8, 8.4], p <.05.<br>**Simon (2017),**<br>**Post-Medicaid Expansion**<br>**Routine check-up:**<br>NS.		**Margerison (2020),**<br>**Post Medicaid Expansion**<br>**Cholesterol check-in last year:**<br>NS.	**Simon (2017),**<br>**Post-Medicaid Expansion**<br>**Flu shot:**<br>NS.

Andersen's domains		Measures of potential access			Measures of realized access				
**Type**	**Determinants/ factors**	**Insurance coverage/type**	**Usual source of care/regular primary care provider**	**Barriers to care, delayed, foregone care, unmet need**	**Utilization (use/non-use or frequency of use)**	**Preventive health visits (annual physical exam/routine checkup in last year)**	**Preventive health screening (blood pressure)**	**Preventive health screening (cholesterol screening)**	**Preventive health screening (Influenza shot)**
		NS (Uninsured);<br>NS (Medicaid);<br>NS (Group);<br>**Group (2014)**<br>NS (Uninsured);<br>NS (Medicaid);<br>NS (Group).<br>**Courtemanche (2019),**<br>**<u>Full ACA (w/ Medicaid)</u>:**<br>9.6%, $p < .001$ (Any insurance);<br>3%, $p < .001$ (Any private);<br>2.3%, $p < .001$ (Employer-sponsored);<br>0.8%, $p < .05$, (Individually purchased);<br>6.7%, $p < .001$ (Medicaid);<br>NS (Other).<br>**Daw (2019)**<br>**<u>Post-Medicaid Expansion</u>**<br>**<u>Years 2 and 3 (Post policy)</u>**<br>**Current insurance status:**<br>-7.4%, 95% CI	**Usual source of care**:<br>NS.<br>**Johnston (2018), No personal doctor:**<br>NS.<br>**Simon (2017),**<br>**<u>Post-Medicaid Expansion</u>**<br>**Have a personal doctor:**<br>NS.<br>**Sommers (2015**),<br>**<u>Post-Medicaid Expansion</u>**<br>**No Personal Physician**:<br>-4.1%, 95% CI<br>[-5.7, -2.5].	**Unmet healthcare need, mental health**:<br>OR = 0.57, 95% CI [0.39, 0.83], $p < .01$ (2015);<br>**Unmet Need, Prescription drugs**:<br>OR = 0.60, 95% CI [0.45, 0.80], $p < .0001$ (2015).<br>**Johnston (2018),**<br>**<u>Post-Medicaid Expansion</u>**<br>**Needed but could not see a doctor due to the cost:**<br>-3.8%, $p < .05$ (All Women < 100% FPL).<br>**Margerison (2020),**<br>**<u>Post-Medicaid Expansion</u>**<br>**Avoided seeking care due to cost:**<br>–7.4%, 95% CI [–12.2, –2.6], $p <.05$.<br>**Simon (2017),**<br>**<u>Post-Medicaid Expansion</u>**<br>**Cost a barrier to care:**					

Andersen's domains		Measures of potential access			Measures of realized access				
**Type**	**Determinants/ factors**	**Insurance coverage/type**	**Usual source of care/regular primary care provider**	**Barriers to care, delayed, foregone care, unmet need**	**Utilization (use/non-use or frequency of use)**	**Preventive health visits (annual physical exam/routine checkup in last year)**	**Preventive health screening (blood pressure)**	**Preventive health screening (cholesterol screening)**	**Preventive health screening (Influenza shot)**
		[–8.6, –6.2] (Uninsured);<br>3.6%, 95% CI [2.5, 4.7] (Medicaid);<br>NS (Private, group);<br>3.1%, 95% CI [2.1, 4.1] (Private, nongroup);<br>NS (Other).<br>**<u>Johnston 2018),</u>**<br>**<u>Post-Medicaid Expansion</u>**<br>**Uninsured:**<br>-13.2%, p < .001 (All Women < 100% FPL).<br>**<u>Jones & Sonfield, 2016),</u>**<br>**<u>Post-Medicaid Expansion</u>**<br>**Currently uninsured:**<br>OR = 2.01, 95% CI [1.64–2.47], p < .001 (Lived in non-expansion state)<br>(Ref = Lived in Medicaid expansion state).<br>**<u>Margerison (2020),</u>**		NS.<br>**Sommers (2015),**<br>**<u>Post Medicaid Expansion</u>**<br>**Cannot Afford Care:**<br>-4.2%, 95% CI<br>[-5.9, -2.5].					

Andersen's domains		Measures of potential access			Measures of realized access				
**Type**	**Determinants/ factors**	**Insurance coverage/type**	**Usual source of care/regular primary care provider**	**Barriers to care, delayed, foregone care, unmet need**	**Utilization (use/non-use or frequency of use)**	**Preventive health visits (annual physical exam/routine checkup in last year)**	**Preventive health screening (blood pressure)**	**Preventive health screening (cholesterol screening)**	**Preventive health screening (Influenza shot)**
		**Post-Medicaid Expansion**<br>9.0%, 95% CI [2.9, 15.2], p <.05 (Has healthcare coverage).<br>**Simon (2017),**<br>**Post-Medicaid Expansion**<br>**Has insurance:**<br>NS.<br>**Sommers (2014),**<br>**Post-Medicaid Expansion**<br>**D.C versus Virginia:**<br>NS (Medicaid);<br>NS (Uninsured);<br>NS (Private insurance);<br>**Connecticut versus other NE States:**<br>1.5%, p < .01 (Medicaid);<br>-2.8%, p < .01 (Uninsured);<br>- 2.6%, p < .01 (Private insurance).<br>**Sommers (2015),**<br>**Post-Medicaid Expansion**							

Andersen's domains		Measures of potential access			Measures of realized access				
Type	Determinants/ factors	Insurance coverage/type	Usual source of care/regular primary care provider	Barriers to care, delayed, foregone care, unmet need	Utilization (use/non-use or frequency of use)	Preventive health visits (annual physical exam/routine checkup in last year)	Preventive health screening (blood pressure)	Preventive health screening (cholesterol screening)	Preventive health screening (Influenza shot)
		**Uninsured**<br>-8.6%, 95% CI<br>[-10.1, -7.0] (Uninsured).<br>**Wehby (2018),**<br>**Post-Medicaid Expansion**<br>6.4%, p < .01 (Medicaid Insurance);<br>-4.7%, p < .01 (Uninsured);<br>NS (Individually Purchased Coverage);<br>NS (Employer-Sponsored Coverage);<br>NS (Any Private Coverage).							
	**Living in Medicaid expansion state**	**Jones & Sonfield, 2016),**<br>**Medicaid expansion (2012 versus 2015)**<br>**Currently uninsured:**<br>OR = 0.50, 95% CI [0.40–0.61], p <. 001 (2015)<br>(Ref = 2012).							
**Contextual Need factors**	**N/A**								

Notes

NS - Non-significant findings.
