# Supplementary material for: Factors affecting women’s access to primary care in the United States since the Affordable Care Act: A mixed-methods systematic review
Source: PLoS One. 2024 Dec 19;19(12):e0314620. doi: 10.1371/journal.pone.0314620 (PMC11658587; doi:10.1371/journal.pone.0314620)
Supplement: S3 Table — (PDF) [file pone.0314620.s007.pdf]

S3 Table. Andersen's model domains and themes relating to facilitators and barriers to access.

Andersen's model domains	Themes	Descriptions	Salient Quotes from participants or excerpts from study findings or discussion
**Facilitators of access**			
**Individual predisposing characteristics**	**Positive health beliefs**	Positive health beliefs about what constitutes health, the cause of illness, and ways to overcome an illness	*"Your relationships . . . your emotional stability, that also influences your body's health. While you are stable in your family, where there is no conflict, and there are no problems… health is going to be okay."* **(Greder & Reina, 2019)**<br>*"I don't think anyone has control [of their own health], but we do have lots of control over our nutrition and taking care of ourselves."* **Participant #8, Mexico (Luque et al., 2018)**<br>*"I believe that if a person is having psychological problems, this will surely affect his physical health. It will affect the blood pressure and also increase the susceptibility for diabetes. That is why I think having good mental health is the most important thing to remain healthy."* **Participant, Focus Group 3 (Ross Perfetti et al., 2019)**
	**Health affirming behaviors**	Behaviors that support health promotion, maintenance, or restoration of health	*"Avoid eating a lot of fat or meat. Like eating vegetables, fruits, drinking a lot of water and everything; do not drink soda . . . take vitamins and all that."* **(Greder & Reina, 2019)**<br>*"I used to be overweight, but since then I decided to walk and walk."* **Participant #27, age 25–29, Mexico. (Luque et al., 2018)**
	**Social support**	Social support from family, friends, and religious institutions such as churches	*"Well, there are obstacles because I don't know how to drive. But, she (daughter) takes me (to the doctor)."* **(Greder & Reina, 2019)**<br>*"When my daughter was little I would go with my husband, and he understands English."* **Participant #17 (Luque et al., 2018)**<br>*"My daughters] translate for me, and sometimes, when I need to go to the doctor, I say: 'When you get home from school, we're going to the doctor' [they say] 'Okay' and they translate for me."* **Participant #30, age 40–44, Mexico (Luque et al., 2018)**
**Individual and contextual enabling characteristics**	**Healthcare safety net (Access to available low-cost or no-cost healthcare)**	Access to services such as available free or low-cost options for healthcare services or prescriptions	*"They [clinic in Chalk] charge you according to your income . . . it is cheap. A visit can cost 15 or 20 dollars. There are many medicines that are generic. For example, a medicine for diabetes for every month can cost 4 dollars."* **(Greder & Reina, 2019)**<br>*"It's very expensive to go to the emergency room. So, for routine health you go to different places where they give you free or low-cost care."* **Participant #23 (Luque et al., 2018)**<br>*"I had to fill an application, and then take it to have it approved, and then it was signed … I was originally being charged $400 and then I ended up with a $130*

Andersen's model domains	Themes	Descriptions	Salient Quotes from participants or excerpts from study findings or discussion
			charge." **Participant #27, age 25–29, Mexico (Luque et al., 2018)**
	**Healthcare organization and delivery**	Access to a usual source of care, type of provider, ease of geographic access, transportation and travel time, appointment-making mechanisms, wait time, and interpreter services.	*"One receives quality and personal attention . . . and above all, the doctor speaks Spanish. That's what makes us feel good in that place."* **(Greder & Reina, 2019)**<br>Half of the women were able to transport themselves to a health care provider either by driving or walking **(Greder & Reina, 2019).**<br>In terms of facilitators to health care, some participants reported that they had not felt discrimination in health care facilities and received interpretation assistance and prescription drug discounts. One participant stated, *"Well, yes they gave me an interpreter."* **Participant #29, age 55–59, Mexico (Luque et al., 2018)**
**Barriers to access**			
**Individual predisposing characteristics**	**Immigrant status and linguistic barriers**	Immigrant status, inability to communicate effectively with healthcare providers	*"The first [barrier] is the language, after that it's not having health insurance and then not having money to pay for the appointments."* **Participant #4 (Luque et al., 2018)**
	**Negative health beliefs**	Negative health beliefs about what constitutes health, the cause of illness, and ways to overcome an illness	Findings in this study are consistent with prior studies (Schwingel et al., 2015; Woodward-Lopez & Flores, 2006) that found Latina immigrant women viewed health in terms of not being ill (theme— absence of illness) and may partially explain why some women delayed using health care preventive services **(Greder & Reina, 2019).**<br>*"The body tells us when we're sick, but sometimes we don't listen."* **Participant #15 (Luque et al., 2018)**
**Individual and contextual enabling characteristics**	**Lack of access to insurance coverage and cost of care (inadequate health care safety net)**	Lack of health insurance coverage and costs associated with healthcare services.	*"When we get sick we have to deal with it or take homemade medicines. . . my mom tells us what to do and we have to do it because if we go to the doctor, how much is it going to cost? We have to use the money to eat and send money to the girls there (Mexico)… That's why many people have died because of illnesses because for not having insurance to go to the doctor to get help"* **(Greder & Reina, 2019).**<br>*"One can afford to pay for the doctor's office visit to know what is wrong with you, but not for the treatment."* **Participant #1, age 35–39, Honduras (Luque et al., 2018)**<br>*"*It's very expensive … just one doctor's visit was $135 and then additionally I had to pay for the medicine. Basically, there went half of my weekly income.*"* **Participant #24, age 40–44, Mexico (Luque et al., 2018)**<br>"I don't have anyone at home to help me and I have 4 kids. The welfare programs provide food stamps and some money. I got $750. How are we supposed to survive? **Participant, Focus Group 2 (Ross Perfetti et al., 2019)**

Andersen's model domains	Themes	Descriptions	Salient Quotes from participants or excerpts from study findings or discussion
	**Healthcare organization and delivery barriers**	Healthcare delivery system characteristics include access to a usual source of care or medical home, type of provider, geographic location, transportation, travel time, appointment-making mechanisms, wait time, and culturally appropriate care such as interpreter services.	*"The first [barrier] is the language, after that it's not having health insurance and then not having money to pay for the appointments."* **Participant #4, age 30–34, Mexico (Luque et al., 2018)**<br>*"Well, that is an obstacle here, there is no public transportation."* **ID #17, age 60–64, Mexico (Luque et al., 2018)**<br>The most salient barrier to receiving healthcare services were problems within healthcare structures. The women cited difficulty contacting the clinic, long wait times, and lack of follow-up with test results **(Ross Perfetti et al., 2019).**<br>The women's reports of not being believed by healthcare providers raise questions about the role of race and gender in determining their experiences of barriers to care, and in particular, the intersection of anti-Muslim and anti-immigrant sentiment with cultural beliefs about health and illness to create barriers to care **(Ross Perfetti et al., 2019).**
**Healthcare behaviors**	**Delayed care**	Delaying or avoiding seeking healthcare.	*"When oneself gets sick there is nothing we can do, if I get sick it will last at least two weeks... I just spread myself with vapor rub or I take ibuprofen and that's it... the money is sometimes what impedes you... like we try to make home medicines at home and then if it is not working then we go to the doctor."* **(Greder & Reina, 2019)**<br>*"Well if something hurts… you wait until you feel really sick and then you run to the doctor. In the meantime, you take a pill or something for the pain."* **Participant #26, age 35–39, Mexico (Luque et al., 2018)**<br>Participants pursued regular visits with health professionals for checkups, screenings, and chronic illness management, however, some reported often avoiding visiting a health professional until symptoms were intolerable because of a variety of barriers to care **(Ross Perfetti et al., 2019).**
	**Health service alternatives**	Shopping for healthcare options	*"I don't like it here [Paris Hills], because they do not attend very well... sometimes one goes to the emergency room and they leave you there waiting for hours then the doctors gets there to see you, so I prefer to travel 45 minutes and once I get there they help me right away."* **(Greder & Reina, 2019)**<br>A common theme that emerged was the idea of "shopping around" from clinic to clinic based on cost, language access and available services to avoid the high cost of emergency room care **(Luque et al., 2018).**
